# Type H vessel/platelet‐derived growth factor receptor β^+^ perivascular cell disintegration is involved in vascular injury and bone loss in radiation‐induced bone damage

**DOI:** 10.1111/cpr.13406

**Published:** 2023-01-24

**Authors:** Jiayan Li, Xiaodan Chen, Lin Ren, Xijuan Chen, Tong Wu, Yun Wang, Xianyue Ren, Bin Cheng, Juan Xia

**Affiliations:** ^1^ Hospital of Stomatology Sun Yat‐sen University Guangzhou China; ^2^ Guangdong Provincial Key Laboratory of Stomatology Sun Yat‐sen University Guangzhou China; ^3^ Guanghua School of Stomatology Sun Yat‐sen University Guangzhou China

## Abstract

Collapse of the microvascular system is a prerequisite for radiation‐induced bone loss. Since type H vessels, a specific bone vessel subtype surrounded by platelet‐derived growth factor receptor β^+^ (PDGFRβ^+^) perivascular cells (PVCs), has been recently identified to couple angiogenesis and osteogenesis, we hypothesize that type H vessel injury initiates PDGFRβ^+^ PVC dysfunction, which contributes to the abnormal angiogenesis and osteogenesis after irradiation. In this study, we found that radiation led to the decrease of both type H endothelial cell (EC) and PDGFRβ^+^ PVC numbers. Remarkably, results from lineage tracing showed that PDGFRβ^+^ PVCs detached from microvessels and converted the lineage commitment from osteoblasts to adipocytes, leading to vascular injury and bone loss after irradiation. These phenotype transitions above were further verified to be associated with the decrease in hypoxia‐inducible factor‐1α (HIF‐1α)/PDGF‐BB/PDGFRβ signalling between type H ECs and PDGFRβ^+^ PVCs. Pharmacological blockade of HIF‐1α/PDGF‐BB/PDGFRβ signalling induced a phenotype similar to radiation‐induced bone damage, while the rescue of this signalling significantly alleviated radiation‐induced bone injury. Our findings show that the decrease in HIF‐1α/PDGF‐BB/PDGFRβ signalling between type H ECs and PDGFRβ^+^ PVCs after irradiation affects the homeostasis of EC‐PVC coupling and plays a part in vascular damage and bone loss, which has broad implications for effective translational therapies.

## Graphical abstract

The interaction of type H vessels and platelet‐derived growth factor receptor β^+^ (PDGFRβ^+^) perivascular cells (PVCs) in the physiological and irradiated microenvironment. In the physiological state, PDGFRβ^+^ PVCs are distributed around type H vessels and participate in osteogenesis. After irradiation, the decrement in hypoxia‐inducible factor‐1α (HIF‐1α)/PDGF‐BB/PDGFRβ signalling between endothelial cells and PVCs leads to the separation of PDGFRβ^+^ PVCs and type H vessels. PDGFRβ^+^ PVCs detach from blood vessels with a osteogenic‐to‐adipogenic transition, which promotes vascular injury, bone loss and adipose accumulation.

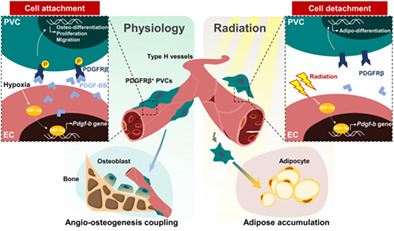

## 1 INTRODUCTION

Due to the high metabolism and calcium content, bone tissue has a higher radiation sensitivity^1^ and absorption rate^2^ than other tissues, resulting in common complications such as osteoradionecrosis, radiation‐induced osteoporosis and refractory pathological fractures after radiotherapy.^3^, ^4^, ^5^ Nevertheless, the pathogenesis of radiation‐induced bone injury has not been fully elucidated, and the efficacy of clinically used interventions and therapies seems to be limited. An in‐depth understanding of the mechanism of radiation‐induced bone injury would pave the way for novel effective targeted therapy.

Radiation can directly injure cells by damaging DNA structure through ionization or inducing the accumulation of reactive oxygen species. It can also exert deleterious effects indirectly by remodelling the cell microenvironment.^6^, ^7^, ^8^ Microvessels are a key component of the bone marrow microenvironment.^9^, ^10^, ^11^ In addition to transporting oxygen and nutrients, endothelial cells (ECs) in blood vessels release a variety of paracrine signals, known as angiocrine signals, to form a unique vascular niche that regulates the self‐renewal and differentiation of perivascular stem and progenitor cells.^11^, ^12^, ^13^ There is growing recognition that radiation deteriorates perivascular mesenchymal stem cells (MSCs) by disrupting their vascular niche^7^, ^14^; however, the underlying mechanism remains largely unknown.

With further research, intraosseous microvessels have been divided into two subtypes, type H vessels (CD31^high^/Emcn^high^) and type L vessels (CD31^low^/Emcn^low^), according to their differences in surface markers, morphology and distribution.^15^ Type H vessels, with high expression of Endomucin (Emcn) and CD31, are located in the metaphysis and participate in bone formation by regulating perivascular osteoprogenitors via paracrine signalling,^16^ whereas type L vessels, which express Emcn and CD31 at a low level, are associated with haematopoiesis.^11^, ^15^ Different microvessel subtypes are surrounded by different types of perivascular cells (PVCs).^17^ Among them, PDGFRβ^+^ PVCs are mainly distributed around osteogenesis‐related type H vessels.^15^, ^16^, ^18^ Previous studies have revealed that pericytes in the bone marrow with osteogenic capacity are PDGFR‐positive,^19^ and PDGFRβ is specific for the osteolineage cluster in single‐cell RNA‐seq and labels skeletal stem and progenitor cells and their reparative progeny during fracture healing,^20^ implying the osteogenic potential of PDGFRβ^+^ PVCs. In soft tissues, such as the brain, retina, heart and kidney, PDGFRβ^+^ PVCs have been reported to function widely as pericytes to maintain vascular stability physiologically. In addition, PDGFRβ^+^ pericytes can detach from microvessels and undergo differentiation transition in pathological conditions.^21^, ^22^, ^23^, ^24^, ^25^, ^26^ However, the biological characteristics of PDGFRβ^+^ PVCs near type H vessels in the bone marrow are not yet clear. Of note, in contrast to type L vessels, type H vessels express a relatively high levels of angiocrine signals with osteogenic properties, including platelet‐derived growth factor‐BB (PDGF‐BB),^11^, ^15^, ^27^, ^28^ which is the main ligand of PDGFRβ. It is speculated that PDGF‐BB secreted by type H ECs plays a role in the regulation of PDGFRβ^+^ PVCs and orchestrate angiogenesis and osteogenesis at the molecular level in the skeletal system.

Here we hypothesize that the change in type H vessels and PDGFRβ^+^ PVCs after irradiation contributes to abnormal angiogenesis and osteogenesis during the progression of bone injury. To address this, we first analysed the characteristic alterations of Type H vessels and PDGFRβ^+^ PVCs in a murine model of local radiation‐induced bone injury and confirmed the changes in PDGFRβ^+^ PVCs with lineage tracing. Then we focused on the changes in EC‐derived PDGF‐BB and its effects on PDGFRβ^+^ PVCs to clarify the role of EC‐PVC interactions in the progression of radiation‐induced bone injury. Finally, we elucidated the experimental therapy for radiation‐induced bone loss based on the revival of hypoxia‐inducible factor‐1α (HIF‐1α)/PDGF‐BB/PDGFRβ signalling between ECs and PDGFRβ^+^ PVCs. Our findings provide proof‐of‐principle for the therapeutic targeting of specific EC‐PVC coupling in the metaphysis in radiation‐induced bone injury.

## 2 RESULTS

### 2.1 Radiation disrupts the homeostasis of type H vessels and PDGFRβ ^+^ PVCs

**FIGURE 1:**
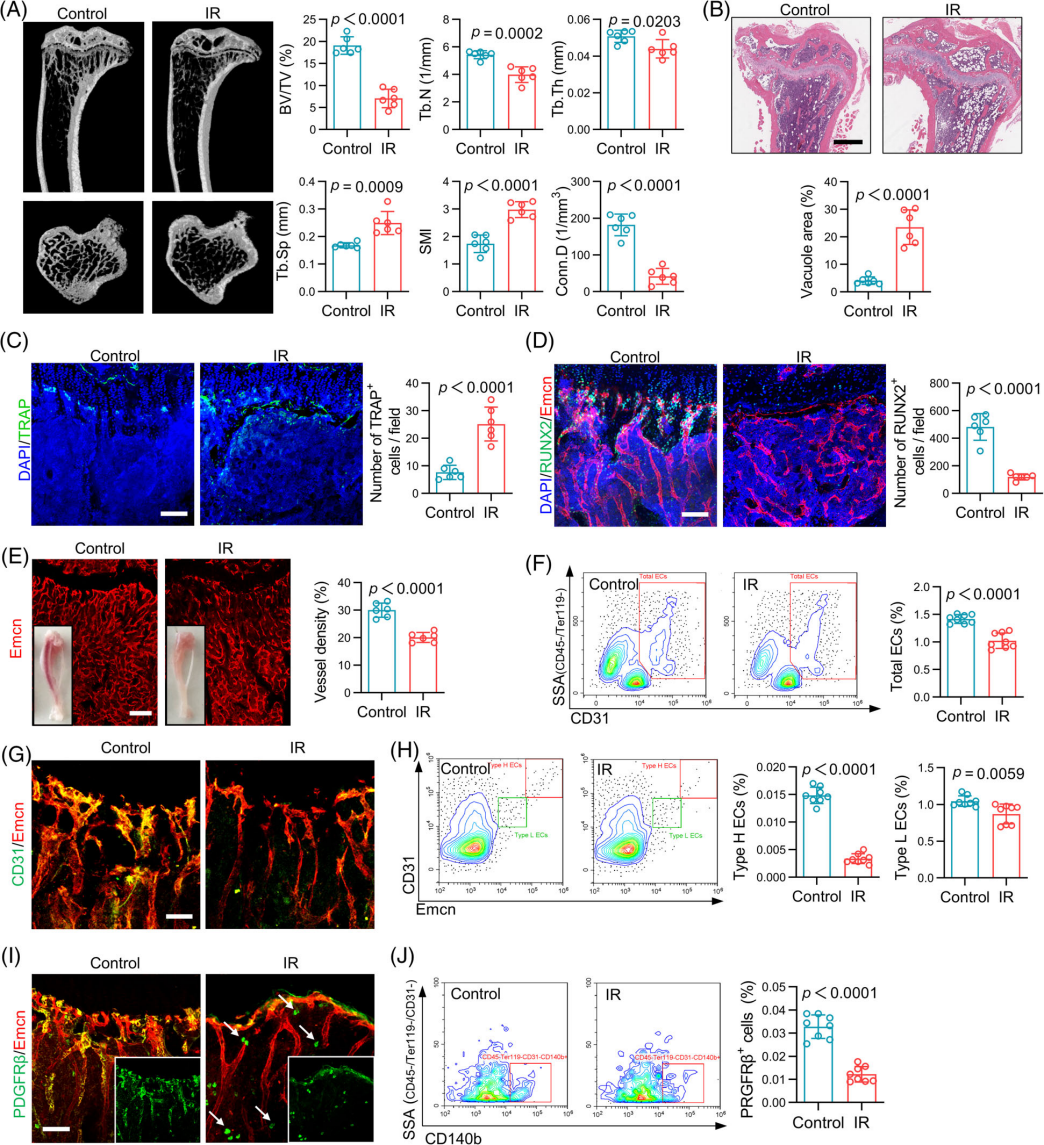
The integration of type H endothelial cells (ECs) and platelet‐derived growth factor receptor β^+^ (PDGFRβ^+^) perivascular cells is disturbed after radiation. (A) Representative micro‐computed tomography images and quantitative cancellous bone parameters of the control and irradiated tibiae (*n* = 6). (B) Representative haematoxylin and eosin staining images of the metaphysis region and quantification of adipocytes of the control and irradiated tibiae. Scale bars, 500 μm (*n* = 6). (C) Representative fluorescence images of the metaphysis region in the control and irradiated tibiae stained with TRAP (green) and DAPI (blue). Quantitative analysis of the number of TRAP^+^ cells. Scale bars, 100 μm (*n* = 6). (D) Representative fluorescence images of the metaphysis region in the control and irradiated tibiae stained with RUNX2 (green), Emcn (red) and DAPI (blue). Quantitative analysis of the number of RUNX2^+^ cells. Scale bars, 100 μm (*n* = 6). (E) Representative anatomic images and corresponding fluorescence images of the control and irradiated tibial sections stained with Emcn (red). Vessel density was quantified according to the fluorescent area. Scale bars, 200 μm (*n* = 6). (F) Flow cytometric quantitation of total ECs (CD31^+^/CD45^−^/Ter119^−^) from the femurs and tibiae of the control and irradiated mice (*n* = 8). (G) Representative fluorescence images of the tibial sections stained with CD31 (green) and Emcn (red) show type H vessels in the metaphysis. Scale bars, 100 μm. (H) Flow cytometric quantitation of type H ECs (CD31^high^/Emcn^high^) and type L ECs (CD31^low^/Emcn^low^) from the femurs and tibiae of the control and irradiated mice (*n* = 8). (I) Representative fluorescence images of tibial sections stained with PDGFRβ (green) and Emcn (red). The white arrows represent PDGFRβ^+^ cells detached from microvessels. Scale bars, 100 μm. (J) Flow cytometric quantitation of PDGFRβ^+^ cells (CD45^−^/Ter119^−^/CD31^−^/CD140b^+^) from the metaphysis region of tibiae of the control and irradiated mice (*n* = 8). Data are represented as the mean ± SD. The *p*‐value was calculated by unpaired, two‐tailed Student's *t*‐test. BV/TV, trabecular bone volume fraction; Tb.N, trabecular number; Tb.Th, trabecular thickness; Tb.Sp, trabecular separation; SMI, structure model index; Conn.D, connectivity density

Not surprisingly, 3D reconstruction of micro‐computed tomography (micro‐CT) and trabecular bone quantification showed a remarkable loss of cancellous bone mass in the metaphysis of the tibiae on day 28 after irradiation (Figure 1A), along with a significant accumulation of adipocytes (Figure 1B). In addition, a marked increase in TRAP^+^ cells (Figure 1C) and a significant decrease in perivascular RUNX2^+^ cells (Figure 1D) were observed in the metaphysis, indicating the loss of the balance between osteogenic and osteoclastic activity. Additionally, the microvessel density and the number of total ECs in bone marrow were reduced (Figure 1E,F), demonstrating the impairment of the microvascular system.

Previous research has shown that the sensitivity of ECs to irradiation varies in different tissues/organs.^14^ Additionally, different EC subtypes in bone marrow also revealed different responses after irradiation. The result of immunofluorescent staining revealed a relatively obvious decline in microvessels in the metaphysis (Figure 1E), which was further confirmed to be the recession of type H vessels with double‐staining of Emcn and CD31 (Figure 1G). Although flow cytometry (FCM) showed an increase in type H ECs (Emcn^high^/CD31^high^) in the incipient stage after irradiation (Figure S1A), it might be due to the upregulation of Emcn and CD31 expression in sinusoid ECs according to previous studies.^15^, ^29^ The DNA damage in the metaphysial ECs was more obvious than that in diaphyseal ECs at 6 h post‐irradiation (Figure S1B), revealing the injury of type H vessels in the metaphysis. As the injury progressed, the number of type H ECs displayed a significant decrease on day 28 after irradiation (Figure 1H), resulting in the deficiency of type H vessels in the metaphysis (Figure 1G). Notably, the falling range of type H ECs was larger than that of type L ECs (Figure 1H), echoing the distinct decline of microvessels in the metaphysis (Figure 1E). Similarly, PDGFRβ^+^ PVCs, which were mainly located close to type H vessels in the nonirradiated tibia (Figure 1I), ranged from increase to decrease in quantitative terms after irradiation (Figures S1C and 1J). Moreover, PDGFRβ^+^ PVCs detached from the metaphysial vessels, resulting in vessel sparseness (Figure 1I). These correlated alterations in number and distribution suggested a close interconnection between PDGFRβ^+^ PVCs and type H vessels.

Overall, irradiation led to impairment of angiogenesis and osteogenesis in the bone marrow. Among these, type H vessels and PDGFRβ^+^ PVCs in the metaphysis reveal correlated alterations. However, the internal mechanism between the ECs and PVCs, as well as the functional change in PDGFRβ^+^ PVCs under the radiation microenvironment, remains to be further explored.

### 2.2 Radiation weakens the vascular stability and osteogenic potential of PDGFRβ;Td^+^ cells

**FIGURE 2:**
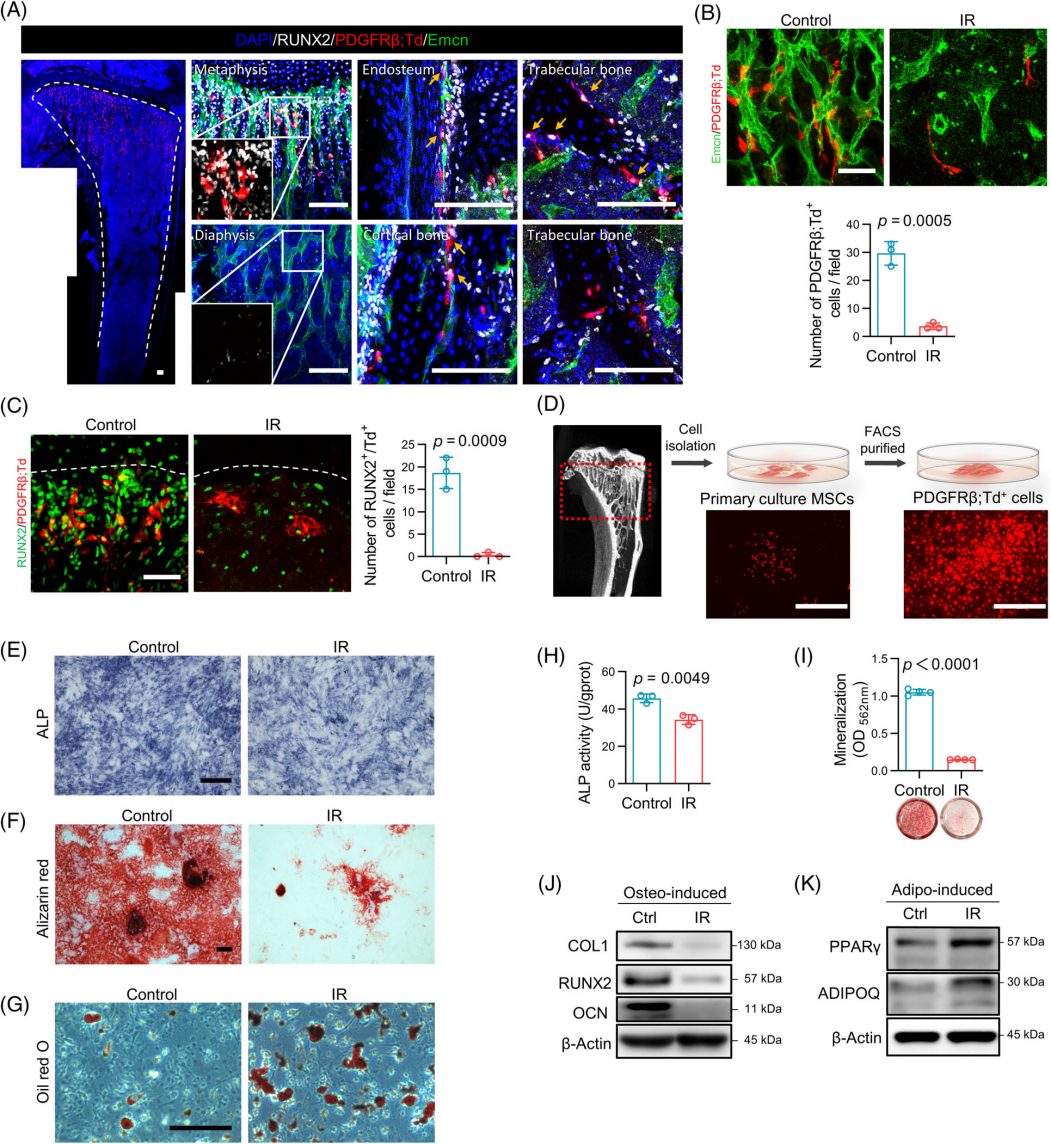
The biological behaviour of platelet‐derived growth factor receptor β (PDGFRβ);Td^+^ cells is changed in the irradiated bone. (A) Representative fluorescence images of tibiae from 8‐week‐old PDGFRβ;Td mice immunostained with Emcn (green), RUNX2 (grey) and DAPI (blue), which show the distribution of PDGFRβ;Td^+^ cells and their osteogenic potential. The yellow arrows represent RUNX2‐positive PDGFRβ;Td^+^ cells. Scale bars, 100 μm. (B) Representative Emcn (green) staining images of the metaphysis region of tibial sections from the control and irradiated PDGFRβ;Td mice reveal the disassembly of microvessels and PDGFRβ;Td^+^ cells. Quantitative analysis of the number of PDGFRβ;Td^+^ cells. Scale bars, 50 μm (*n* = 3). (C) Representative RUNX2 (green) staining images and quantitative analysis of the number of RUNX2^+^/Td^+^ cells of the metaphysis region of tibial sections from the control and irradiated PDGFRβ;Td mice reveal the disappearance of RUNX2‐positive PDGFRβ;Td^+^ cells. White dashed lines represent the bottom of the growth plate. Scale bars, 50 μm (*n* = 3). (D) Diagram and representative fluorescence images of the isolation and purification of PDGFRβ;Td^+^ cells from the metaphysis region of PDGFRβ;Td mice. Scale bars, 1000 μm. (E–G) Representative image of alkaline phosphatase (ALP) staining (E), Alizarin red staining (F) and Oil red O staining (G) of PDGFRβ;Td^+^ cells isolated from the control and irradiated mice. Scale bars, 200 μm. (H) Relative ALP activity of PDGFRβ;Td^+^ cells isolated from the control and irradiated mice (*n* = 3). (I) Semi‐quantitative analysis of Alizarin red staining of PDGFRβ;Td^+^ cells isolated from the control and irradiated mice (*n* = 3). (J) Western blot analysis of osteogenic proteins including COL1, RUNX2 and OCN in PDGFRβ;Td^+^ cells from the control and irradiated mice. (K) Western blot analysis of adipogenic proteins including PPARγ and ADIPOQ in PDGFRβ;Td^+^ cells from the control and irradiated mice. Data are represented as the mean ± SD. The *p*‐value was calculated by unpaired, two‐tailed Student's *t*‐test. FACS, fluorescence‐activated cell sorting; MSCs, mesenchymal stem cells

Given that PDGFRβ labels the osteolineage cluster in bone marrow,^19^, ^20^ it is reasonable to assume that PDGFRβ^+^ PVCs represent a subset of osteogenic lineage cells near type H vessels. To confirm this, PDGFRβ‐CreER^T2^;Rosa26‐LSL‐Tdtomato (PDGFRβ;Td) mice were generated, in which Tdtomato was induced in PDGFRβ‐expressing cells and their progeny after tamoxifen administration. According to the immunofluorescent staining of tibial sections, a majority of the PDGFRβ;Td^+^ cells were distributed near type H vessels in the metaphysis, but not adjacent to type L vessels in the diaphyseal. Additionally, most PDGFRβ;Td^+^ PVCs showed positive expression of the classic osteogenic marker RUNX2. Moreover, RUNX2^+^/PDGFRβ;Td^+^ cells could also be found adjacent to the metaphysial microvessels, in the endosteum, inside the cortical bone, and on the surface of the trabeculae. Some of the PDGFRβ;Td^+^ cells were even embedded in the mature trabeculae (Figure 2A), confirming the osteogenic lineage of PDGFRβ;Td^+^ cells and their competency in bone formation. However, PDGFRβ;Td^+^ PVCs were significantly decreased in the irradiated metaphysis, along with the unordered and sparse microvessels. The remaining Td^+^ cells detached from microvessels and no longer expressed RUNX2 (Figure 2B,C), demonstrating the decrease in vascular stability and osteogenic potential in PDGFRβ;Td^+^ cells after irradiation.

Next, to further verify the change in differentiation potential in PDGFRβ;Td^+^ cells, we isolated bone marrow MSCs and purified PDGFRβ;Td^+^ cells by fluorescence‐activated cell sorting (FACS; Figure 2D). After in vitro induction, PDGFRβ;Td^+^ cells displayed osteogenic and adipogenic differentiation abilities (Figure S2A,B), which is one of the characteristics of MSCs. Notably, the osteogenic differentiation potential of PDGFRβ;Td^+^ MSCs was stronger than that of PDGFRβ;Td^−^ MSCs both with and without PDGF‐BB stimulation after 14 days of osteogenic induction (Figure S2C), implying that the PDGFRβ^+^ subset might play an important role in osteogenesis when compared with the PDGFRβ^−^ subset. However, PDGFRβ;Td^+^ cells from the irradiated mice displayed a significant reduction in osteogenic potential and an elevation in adipogenic potential according to the results of alkaline phosphatase (ALP) evaluation, matrix mineralization staining and lipid droplet staining (Figure 2E–I). In addition, PDGFRβ;Td^+^ cells exhibited downregulation of osteogenic markers and upregulation of adipogenic markers in the irradiated group (Figure 2J,K), revealing a functional shift from osteogenic to adipogenic.

Combining the in vivo and in vitro results, we found the perivascular decline of PDGFRβ;Td^+^ cells as well as the lineage transition of the remaining Td^+^ cells were involved in vascular damage and the imbalance of osteogenic and adipogenic activity after irradiation.

### 2.3 Radiation diminishes EC‐derived PDGF‐BB and affects PDGFRβ;Td^+^ PVCs indirectly

Radiation exerts deleterious effects on bone marrow cells directly and indirectly. Unlike some of the metaphysial ECs showed H2A.X‐positive after irradiation (Figure S1B), PDGFRβ^+^ cells showed inconspicuous DNA damage in the irradiated tibia (Figure S3A). Besides, in contrast to human microvascular endothelial cell line‐1 (HMEC‐1), whose proliferation can be significantly inhibited by direct irradiation in vitro, the primary cultured MSCs, whether Td positive or not, revealed relatively resistance to a high dose of direct radiation exposure (Figure S3B–D). This evidence demonstrated the relative radioresistance of PDGFRβ;Td^+^ cells and suggested that the biological changes in Td^+^ cells after irradiation were not only dependent on the direct effects of radiation.

According to the perivascular character of PDGFRβ;Td^+^ PVCs, angiocrine signals seem to be one of the major indirect influencing factors for the alteration of the cells. Previous studies have shown that angiocrine signal PDGF‐BB, which is the main ligand of PDGFRβ, play an important role in the regulation of proliferation, migration and differentiation of perivascular PDGFRβ^+^ cells.^30^ Given this evidence, we propose that the changes in PDGFRβ^+^ PVCs after irradiation may be relevant to EC‐derived PDGF‐BB. To address this, we next detected the expression and distribution of PDGF‐BB in irradiated bone.

**FIGURE 3:**
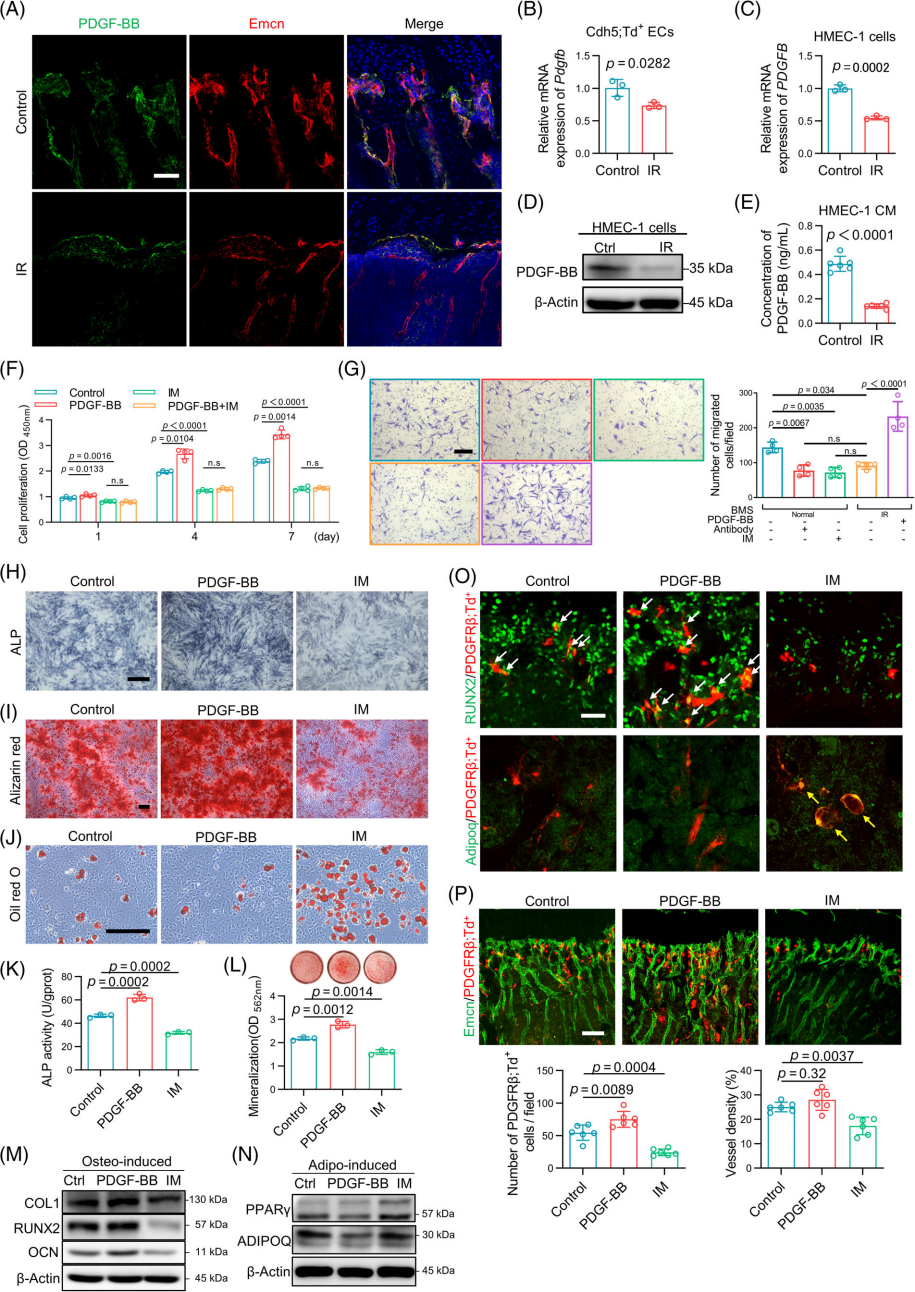
The alterations of platelet‐derived growth factor receptor β (PDGFRβ);Td^+^ cells after radiation are associated with endothelial cell (EC)‐derived PDGF‐BB. (A) Representative fluorescence images of the control and irradiated tibial sections stained with PDGF‐BB (green), Emcn (red) and DAPI (blue). Scale bars, 50 μm. (B,C) Quantitative real‐time PCR analysis of the Pdgfb gene in Cdh5;Td^+^ ECs (B) and human microvascular endothelial cell line‐1 (HMEC‐1) cells (C; *n* = 3). (D) Western blot analysis of PDGF‐BB in the control and irradiated HMEC‐1 cells. (E) Enzyme‐linked immunosorbent assay analysis of PDGF‐BB in the conditioned medium (CM) of the control and irradiated HMEC‐1 cells (*n* = 6). (F) Cell Counting Kit‐8 assay of the PDGFRβ;Td^+^ cells after PDGF‐BB and/or imatinib mesylate (IM) treatment (*n* = 4). (G) Migration assay of PDGFRβ;Td^+^ cells in the bone marrow supernatant collected from the nonirradiated or irradiated bone. Td^+^ cells received additional in vitro intervention with PDGF‐BB neutralizing antibody, IM, or recombinant PDGF‐BB protein. Scale bars, 200 μm. (*n* = 4). (H–J) Representative images of alkaline phosphatase (ALP) staining (H), Alizarin red staining (I) and Oil red O staining (J) of PDGFRβ;Td^+^ cells treated with PDGF‐BB or IM during osteogenic/adipogenic induction. Scale bars, 200 μm. (K) Relative ALP activity of PDGF‐BB‐treated or IM‐treated PDGFRβ;Td^+^ cells (*n* = 3). (L) Semiquantitative analysis of Alizarin red staining of PDGF‐BB‐treated or IM‐treated PDGFRβ;Td^+^ cells (*n* = 3). (M,N) Western blot analysis of osteogenic‐associated protein (M) and adipogenic‐associated protein (N) in PDGFRβ;Td^+^ cells treated with PDGF‐BB or IM during osteogenic/adipogenic induction. (O) Representative RUNX2 or Adipoq staining images of the metaphysis region of tibial sections from the PDGFRβ;Td mice. PDGF‐BB and IM were administered via intra‐tibial injection. The white and yellow arrows represent RUNX2^+^/PDGFRβ;Td^+^ cells and Adipoq^+^/PDGFRβ;Td^+^ cells, respectively. Scale bars, 25 μm. (P) Representative Emcn (green) staining images and quantification of PDGFRβ;Td^+^ cells and vessel density in the metaphysis region of the tibial sections from PDGF‐BB‐treated or IM‐treated PDGFRβ;Td mice. Scale bars, 100 μm. Data are represented as the mean ± SD. The *p*‐value was calculated by unpaired, two‐tailed Student's *t*‐test when comparing two treatment groups and by one‐way analysis of variance with Tukey's post hoc test when comparing multiple groups. BMS, bone marrow supernatant

As previously reported, PDGF‐BB in bone marrow was largely derived from ECs and preosteoclasts.^20^, ^31^ Here, the results of immunofluorescence staining showed a high degree of overlap of the endothelial marker Emcn and PDGF‐BB in the metaphysis of the nonirradiated tibia (Figure 3A), but not TRAP^+^ preosteoclasts (Figure S4A), indicating that the metaphysial ECs, namely, type H ECs according to morphology and distribution, were the major source of PDGF‐BB in the metaphysis. After irradiation, the synthesis and secretion levels of PDGF‐BB declined in the whole bone tissue (Figure S4B,C). Correspondingly, the expression of PDGF‐BB in the metaphysial ECs was reduced significantly in the irradiated tibia (Figure 3A). To exclude the possibility that the downregulation of PDGF‐BB after irradiation was mainly due to the decrease in EC number, Cdh5;Td^+^ ECs in vivo and HMEC‐1 cells in vitro were used to quantify the expression level of PDGF‐BB in total ECs after irradiation. The results showed that the normalized expression of PDGF‐BB in irradiated ECs was significantly downregulated at the transcriptional, translational and secreted levels (Figure 3B–E). Of note, we found that the phosphorylation level of PDGFRβ was significantly decreased in PDGFRβ;Td^+^ cells from the irradiated mice (Figure S5A). PDGF‐BB is the main activator of PDGFRβ phosphorylation.^30^, ^32^, ^33^ In vitro supplementation with recombinant PDGF‐BB protein and PDGFRβ inhibitor imatinib mesylate (IM) increased and decreased the phosphorylation level of PDGFRβ in Td^+^ cells, respectively (Figure S5B). Thus, the decrease in PDGFRβ phosphorylation after irradiation verified the decline in PDGF‐BB again and revealed the downregulation of PDGF‐BB/PDGFRβ signalling in PDGFRβ;Td^+^ cells.

Next, we activated or inhibited PDGF‐BB/PDGFRβ signalling in vitro and in vivo to determine whether its effect on PDGFRβ;Td^+^ cells was similar to that caused by irradiation. Unsurprisingly, when PDGFRβ was blocked by IM, the proliferation ability of PDGFRβ;Td^+^ cells was inhibited (Figure 3F). In addition, both irradiation and PDGF‐BB/PDGFRβ signalling interruption significantly reduced the chemotaxis of PDGFRβ;Td^+^ cells in either bone marrow supernatant (BMS) or HMEC‐1 conditioned medium, while the weakened recruit effect of the supernatant from irradiated mice or ECs could be rescued by exogenous PDGF‐BB (Figures 3G and S6).

In addition, recombinant PDGF‐BB protein promoted osteogenic differentiation and inhibited adipogenic differentiation of PDGFRβ;Td^+^ cells both in vitro and in vivo, whereas IM converted the differentiation potential from osteogenic to adipogenic, similar to the performance of Td^+^ cells showed after irradiation. (Figure 3H–O). Besides, the in vivo results also showed that the quantity of PDGFRβ;Td^+^ cells was expanded in the PDGF‐BB treatment group and shrunk in the IM treatment group, and IM treatment also displayed a decrease in microvessel density in the metaphysis, which was similar to those that appeared in the irradiated bone (Figure 3P).

In general, irradiation decreased the expression of PDGF‐BB in metaphysial ECs and led to the downregulation of PDGF‐BB/PDGFRβ signalling in PDGFRβ^+^ PVCs, which not only affected the biological performance of Td^+^ cells themselves but also disturbed vascular homeostasis since PDGFRβ^+^ PVCs could function as pericytes to maintain the stability of the type H vessels.

### 2.4 Exogenous PDGF‐BB relieves radiation‐induced bone injury

**FIGURE 4:**
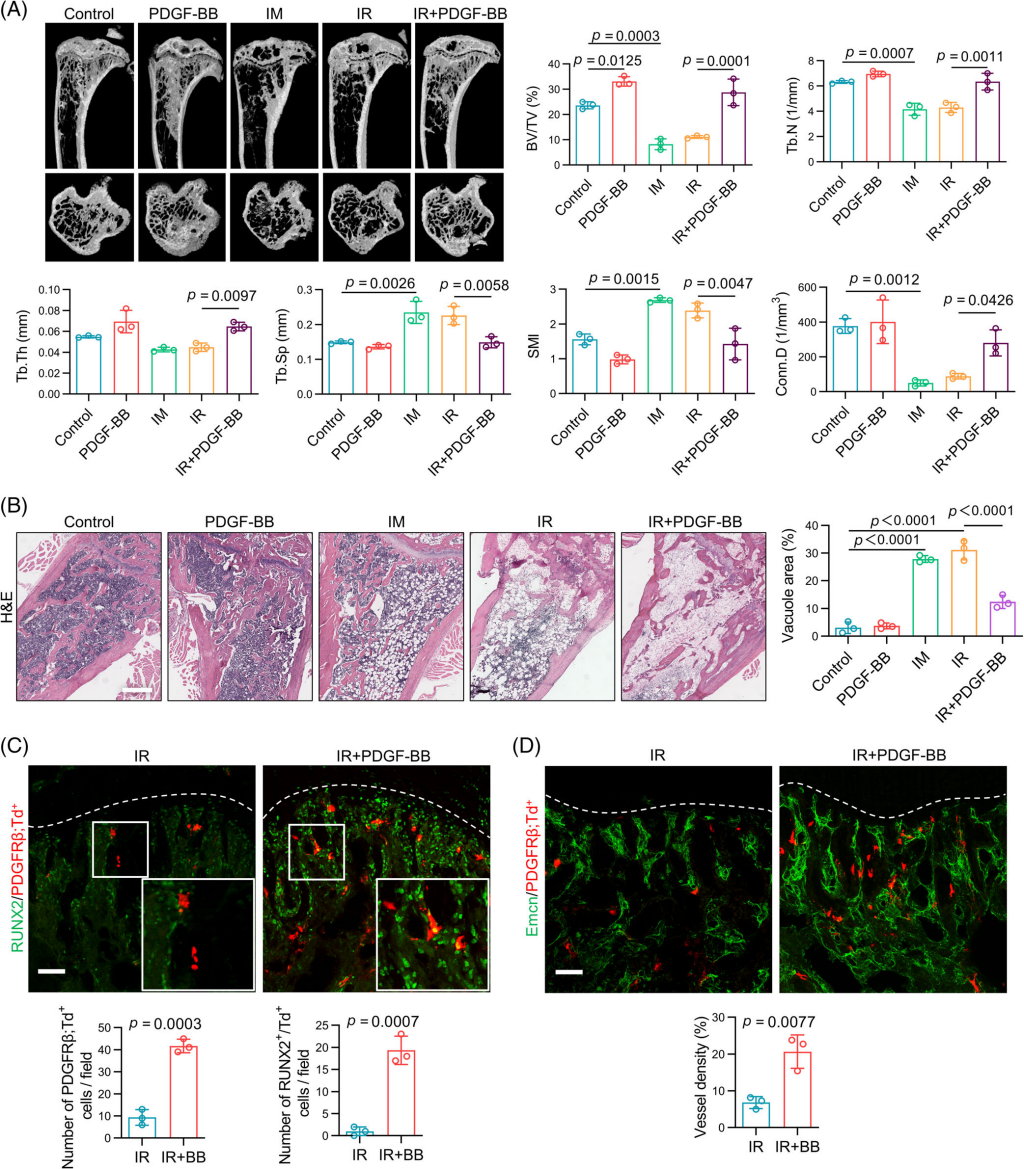
Upregulation of platelet‐derived growth factor (PDGF)‐BB/platelet‐derived growth factor receptor β (PDGFRβ) signalling relieves radiation‐induced bone injury. (A) Representative micro‐computed tomography images and quantitative cancellous bone parameters of the non‐irradiated or irradiated tibiae treated with PDGF‐BB or imatinib mesylate (IM) via intra‐tibial injection (*n* = 3). (B) Representative haematoxylin and eosin (H&E) staining images and quantification of adipocytes of the non‐irradiated or irradiated tibiae treated with intra‐tibial injection as indicated. Scale bars, 500 μm (*n* = 3). (C,D) Representative RUNX2 (C) and Emcn (D) staining images of tibial sections from irradiated PDGFRβ;Td mice with or without intra‐tibial PDGF‐BB injection. Quantitative analysis of the number of PDGFRβ;Td^+^ cells, RUNX2^+^/Td^+^ cells and vessel density. White‐dashed lines represent the bottom of the growth plate. Scale bars, 50 μm (*n* = 3). Data are represented as the mean ± SD The *p*‐value was calculated by unpaired, two‐tailed Student's *t*‐test when comparing two treatment groups and by one‐way analysis of variance with Tukey's post hoc test when comparing multiple groups.

Since the downregulation of PDGF‐BB/PDGFRβ signalling has been proven to be responsible for the functional alteration of PDGFRβ;Td^+^ cells, we then proceeded to elucidate whether it is related to the changes in bone phenotype after irradiation and tried to use exogenous recombinant PDGF‐BB protein as a radio‐protector of bone tissue for rescue therapy. The results of micro‐CT and histological staining showed that the administration of IM, a PDGFRβ inhibitor to block PDGF‐BB/PDGFRβ signalling, could lead to a decline in bone mass and an increase in adipogenesis in the bone marrow that was similar to the phenotypes that appeared in irradiated bone. In contrast, exogenous PDGF‐BB effectively enhanced bone mass (Figure 4A), increased collagen deposition (Figure S7), and suppressed fat accumulation (Figure 4B) in the irradiated tibia. Moreover, PDGFRβ;Td^+^ cells in the metaphysis region showed an increase in numbers and upregulation of RUNX2 expression in the PDGF‐BB treatment group, revealing the maintenance of cell viability and osteogenic potential (Figure 4C). Additionally, vascular disorder after irradiation was alleviated with the administration of PDGF‐BB (Figure 4D).

In short, these results indicated that PDGF‐BB treatment had a certain therapeutic effect on radiation‐induced bone damage, accompanied by the maintenance of PDGFRβ;Td^+^ cell function and vascular homeostasis. The downregulation of PDGF‐BB/PDGFRβ signalling in PDGFRβ;Td^+^ cells, at least in part, was responsible for the bone injury after irradiation.

### 2.5 Upregulation of HIF‐1α/PDGF‐BB/PDGFRβ signalling between ECs and PDGFRβ ^+^ PVCs alleviates radiation‐induced vascular damage and bone loss

**FIGURE 5:**
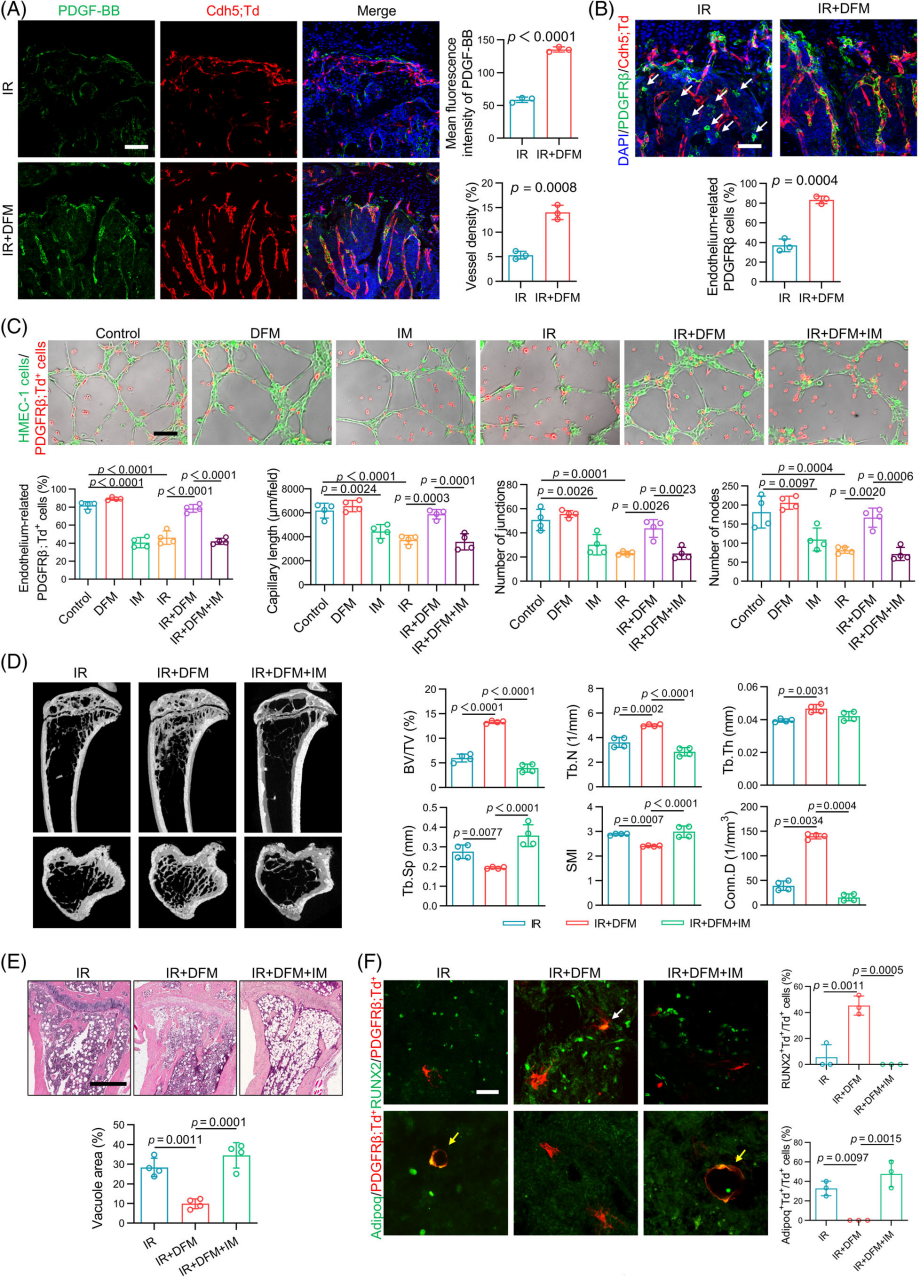
Activation of the endothelial cell (EC)‐perivascular cell axis improves angiogenesis and osteogenesis. (A) Representative platelet‐derived growth factor (PDGF)‐BB (green) staining images of the metaphysis regions of tibial sections from the irradiated Cdh5;Td mice with or without deferoxamine mesylate (DFM) treatment. Nuclei, DAPI (blue). Quantification of fluorescence intensity of PDGF‐BB and vessel density. Scale bars, 100 μm (*n* = 3). (B) Representative PDGF receptor β (PDGFRβ; green) staining images and quantification of vessel‐related PDGFRβ^+^ cells of the metaphysis regions of tibial sections from the irradiated Cdh5;Td mice with or without DFM treatment. Nuclei, DAPI (blue). The white arrows represent detached PDGFRβ^+^ cells. Scale bars, 50 μm (*n* = 3). (C) Representative fluorescence images of human microvascular endothelial cell line‐1 (HMEC‐1) cells (green) and PDGFRβ;Td^+^ cells (red) on Matrigel with quantification of EC‐attached PDGFRβ;Td^+^ cells and tube formation parameters. Scale bars, 50 μm (*n* = 4). (D) Representative micro‐computed tomography (micro‐CT) images and quantitative analysis of the irradiated tibiae with DFM and/or imatinib mesylate (IM) treatment (*n* = 4). (E) Representative haematoxylin and eosin (H&E) staining images and quantification of adipocytes of the irradiated tibiae with DFM and/or IM treatment. Scale bars, 500 μm (*n* = 4). (F) Representative RUNX2 or Adipoq staining images and quantification of the double positive cells in total Td^+^ cells of tibial sections from the PDGFRβ;Td mice. DFM or IM was administered via intra‐peritoneal injection. The white and yellow arrows represent RUNX2^+^/PDGFRβ;Td^+^ cells and Adipoq^+^/PDGFRβ;Td^+^ cells, respectively. Scale bars, 25 μm (*n* = 3). Data are represented as the mean ± SD The *p* value was calculated by unpaired, two‐tailed Student's *t*‐test when comparing two treatment groups and by one‐way analysis of variance with Tukey's post hoc test when comparing multiple groups

Next, we explored the underlying mechanisms by which radiation reduced PDGF‐BB expression in ECs. HIF‐1α is one of the major upstream regulators of PDGF‐BB.^18^, ^34^ Here, we found that the expression of HIF‐1α in the metaphysis was attenuated significantly after irradiation (Figure S8A). However, when deferoxamine mesylate (DFM), an activator of HIF‐1α,^35^, ^36^ was applied, the expression of HIF‐1α was upregulated (Figure S8A,B). Unsurprisingly, the expression of PDGF‐BB in ECs and BMS was also increased (Figures 5A and S8C,D), accompanied by a decrease in detached PDGFRβ^+^ PVCs (Figure 5B). Moreover, the sparse and jumbled microvessels in the metaphysis of the irradiated tibia became columnar and well organized after DFM administration (Figure 5A,B). Similarly, the in vitro results of the co‐culture assay on Matrigel revealed that DFM‐treated HMEC‐1 cells became more attractive to PDGFRβ;Td^+^ cells, but this reinforced chemotaxis could be interrupted when PDGFRβ was pre‐blocked by IM. Concomitantly, the tube formation ability of the irradiated HMEC‐1 cells as well as the PDGFRβ;Td^+^ cell coverage of tubes could be enhanced by DFM but disturbed by the PDGFRβ inhibitor (Figure 5C). These results preliminarily proved that DFM induced the interaction between ECs and PDGFRβ^+^ cells and promoted microvascular stability by enhancing HIF‐1α/PDGF‐BB/PDGFRβ signalling.

Additionally, DFM significantly increased bone mass and decreased bone marrow adipocytes of the irradiated tibia (Figure 5D,E). Correspondingly, the differentiation potential of PDGFRβ;Td^+^ cells was converted with the disappearance of Adipoq^+^/PDGFRβ;Td^+^ cells and the re‐emergence of RUNX2^+^/PDGFRβ;Td^+^ cells in the DFM treatment group (Figure 5F). However, these positive effects of DFM could be counteracted by the PDGFRβ inhibitor (Figure 5D–F). These findings indicated that the activation of PDGF‐BB/PDGFRβ signalling between ECs and PDGFRβ^+^ PVCs was one of the prerequisites for DFM to promote osteogenesis. In short, DFM treatment increased HIF‐1α/PDGF‐BB in ECs and optimized the PDGF‐BB/PDGFRβ signalling between ECs and PDGFRβ^+^ PVCs. In this instance, PDGFRβ^+^ PVCs could act as pericytes to maintain vascular stability and display as osteogenic lineage cells to reverse the imbalance of bone formation and resorption.

These results showed that HIF‐1α/PDGF‐BB/PDGFRβ signalling between ECs and PDGFRβ^+^ PVCs was attenuated in the irradiation microenvironment, which caused both vascular injury and bone loss. Revival of HIF‐1α/PDGF‐BB/PDGFRβ signalling by DFM orchestrates angiogenesis and osteogenesis to alleviate radiation‐induced bone injury.

## 3 DISCUSSION

Knowledge concerning the crucial role of type H vessels and PDGFRβ^+^ PVCs in bone repair and regeneration is increasing rapidly. Thus far, however, little is known about their spatial and temporal crosstalk in the progression of radiation‐induced bone injury. Our present study bridges this gap and reveals that PDGF‐BB deficiency in type H vessels is responsible for the functional changes in PDGFR^+^ PVCs, which participate in vascular injury and bone loss under irradiation. This concept is supported by the following observations: (1) Radiation leads to the decrease in both type H vessels and PDGFRβ^+^ PVC numbers, and the recruitment of PDGFRβ^+^ PVCs by type H vessels is weakened, resulting in the detachment of PDGFRβ^+^ PVCs and further vascular disorder. (2) PDGFRβ^+^ PVCs are away from the original vascular niche and undergo lineage shifts from osteoblasts toward adipocytes, which aggravates bone loss after radiation. (3) The functional alteration of PDGFRβ^+^ PVCs after radiation is associated with the decrease in HIF‐1α/PDGF‐BB/PDGFRβ signalling between ECs and PVCs. (4) Pharmacological blockade of HIF‐1α/PDGF‐BB/PDGFRβ signalling between ECs and PDGFRβ^+^ PVCs induces a phenotype similar to radiation‐induced bone damage, while the rescue of the signalling significantly alleviates vascular disorder and bone injury.

Microvascular lesions have been proposed as an initial factor in the development of radiation‐induced bone injury,^1^, ^7^, ^37^ which is thought to be related to the low tolerance of ECs to radiation.^24^, ^25^, ^38^ Our data further show type H vessels, which were located in the active metaphysis, display more obvious DNA damage and have a larger percentage reduction than type L vessels in the diaphysis. This might be because metabolically active tissues and cells are more sensitive to direct ionizing damage.^6^ Additionally, PDGFRβ^+^ pericyte‐deficient mice demonstrated significant microvessel regression and micro‐circulation failure,^26^, ^39^, ^40^ revealing that PDGFRβ^+^ PVCs play an important role in microvascular homeostasis. Thus, the decline in PDGFRβ^+^ PVCs and their detachment from type H vessels after irradiation also aggravate vascular instability and collapse. In summary, the remarkable reduction in type H vessels in the irradiated tibia is associated with their low radiation tolerance, high metabolic level and homeostatic disturbance caused by the reduction and detachment of PDGFRβ^+^ PVCs.

In contrast to type H ECs, PDGFRβ^+^ PVCs, which share a similar immunophenotype and differentiation potential as MSCs/progenitors,^41^, ^42^, ^43^, ^44^ are more resistant to direct radiation damage. Although PDGFRβ^+^ cells display a similar decreasing amplitude to type H ECs in vivo, cells undergoing high doses of direct radiation still survive and maintain proliferative capacity in vitro, indicating that direct ionizing damage is not the only reason to be blamed, and the indirect influences from the vascular niche are also responsible for the alterations of PDGFRβ^+^ PVCs in the radiation microenvironment. According to the close spatial and functional connection between type H vessels and PDGFRβ^+^ PVCs, we hypothesize that this indirect effect is associated with the paracrine alteration of type H ECs. Among the paracrine factors, PDGF‐BB, which is highly expressed in type H ECs,^27^, ^28^ has been proven to be the major EC‐derived factor that regulates pericyte function.^45^ Here we extend the findings that the PDGF‐BB decline in type H vessels acts as an important trigger for the functional alteration of PDGFRβ^+^ PVCs after radiation, including the reduction in cell number, the abnormality in cell migration and the change in cell differentiation.

In contrast to the decrease of ECs and PDGFRβ^+^ PVCs, we found that the number of osteoclasts in metaphysis increased after irradiation, coinciding with the enhancement of bone loss. However, in addition to the classic bone‐associated osteoclasts which were the major subtype occupying ageing bones, a novel subtype of osteoclasts, vessel‐associated osteoclasts (VAO), were found closely associated with type H vessels and were predominant in early developing bones.^46^ Some key osteoclastogenic factors such as *Csf1*, *Il1a* and *Tnfsf11a* were highly expressed in type H compared with type L ECs, suggesting the considerable crosstalk between type H ECs and VAOs. Interestingly, the number of VAOs increased in the early stage after irradiation,^46^ but the role of the increased VAOs in the progression of bone injury and whether the proportion of different osteoclast subtypes would change in the later period after irradiation were not clear. Besides, we know that bone remodelling is tightly regulated by crosstalk between bone‐forming osteoblasts and bone‐resorbing osteoclasts.^47^ Thus, the interaction between different osteoclasts subtypes and perivascular osteogenic lineage cells under the irradiated micro‐environment is also an interesting topic of research value, which deserves further investigation.

PDGF‐BB/PDGFRβ signalling is one of the most critical pericyte‐centred pathways that are essential for angiogenesis.^48^ Further investigations emphasize its important role in pericyte recruitment to the vessels. In bone, EC‐derived PDGF‐BB aggregates around blood vessels under the action of heparan sulphate, forming a locally high concentration within the vascular niche and attracting PDGFRβ^+^ cells via high‐affinity ligand–receptor interactions.^30^ PDGF‐B‐deficient (*Pdgfb* ^−/−^) mice,^49^, ^50^ PDGF‐B retention‐motif knockout (*Pdgfb* ^*ret/ret*^) mice, which lack perivascular deposits of PDGF‐BB,^51^, ^52^ and endothelial‐specific *Pdgfb* knockout (*Pdgfb^iΔEC^*) mice^53^ all showed reduced coverage of PDGFRβ^+^ pericytes on the vessel surface, indicating the importance of PDGF‐BB in the recruitment of PDGFRβ^+^ cells. However, except for ECs, PDGF‐BB can also be expressed in osteoclast lineage cells and tends to increase in pathological states, thus changing the physiological distribution of PDGF‐BB and switching the migratory route of PDGFRβ^+^ cells. For instance, the expression of PDGF‐BB in TRAP^+^ preosteoclasts was significantly increased in ageing mice and high‐fat diet (HFD)‐fed mice.^54^ Additionally, mononuclear preosteoclasts expressed high levels of PDGF‐BB in a murine model of arthritis, which recruited pericytes for neovessel formation by activating PDGF‐BB/PDGFRβ signalling.^55^ Similarly, in a murine model of spinal cord injury, M2 macrophages stimulated the migration of PDGFRβ^+^ pericytes toward the injury site by secreting PDGF‐BB.^56^ In adipose tissue, both upregulation of PDGF‐BB in M1 macrophages after HFD and administration of exogenous PDGF‐BB induced detachment of perivascular PDGFRβ^+^ cells from the original vascular surface.^57^ These observations indicated that the changes in PDGF‐BB concentration and distribution have a significant impact on the migration of PDGFRβ^+^ PVCs. However, we found that the change in PDGF‐BB expression in the metaphysis after irradiation seems to be related to type H vessels rather than TRAP^+^ preosteoclasts. Interestingly, although PDGF‐BB declines in most metaphysial ECs after radiation, it can still be detected around the residual microvessels tightly below the growth plate, where certain PDGFRβ^+^ PVCs are maintained. Briefly, these findings demonstrate that the decrease of EC‐derived PDGF‐BB in type H ECs in the metaphysis is largely responsible for the detachment of PDGFRβ^+^ PVCs after irradiation.

The osteogenic lineage of PDGFRβ^+^ cells in bone marrow has been revealed in previous studies. Specifically, NG2;Td^+^ pericytes which are PDGFRβ‐positive contribute to bone fracture healing as a cellular source of osteogenic cells.^19^ Single‐cell sequencing revealed that PDGFRβ specifically labelled the osteolineage cluster and marked all osterix^+^ skeletal stem and progenitor cells and their reparative progeny during fracture healing.^20^ On that basis, our study further shows that perivascular PDGFRβ;Td^+^ cells highly express classic osteogenic markers and can be found in bone surface and bone lacuna. Moreover, it has a stronger osteogenic differentiation potential than the PDGFRβ;Td^−^ subset in vitro, highlighting the vital role of PDGFRβ;Td^+^ cells in osteogenesis. However, PDGFRβ;Td^+^ cells tend to differentiate into adipocytes in irradiated bone, reflecting their multi‐directional differentiation ability and their contributions to bone loss and marrow adiposity under radiation. In bone, the differentiation of PDGFRβ^+^ cells is regulated at least partially by PDGF‐BB/PDGFRβ signalling. PDGF‐BB is an FDA‐approved bone repair factor for clinical use and has been demonstrated to be effective in enhancing bone formation.^58^ Studies have shown that PDGF‐BB is involved in the differentiation of pericytes into osteoprogenitor cells and osteoblasts in vivo.^59^ In addition, PDGFRβ signalling activation in pericytes^60^ or exogenous PDGF‐BB administration in adipose‐derived stem cells^61^ could suppress adipogenic differentiation of cells, while the deletion of PDGFRβ enhanced adipogenesis,^62^ identifying PDGF‐BB/PDGFRβ signalling deficiency as a motivator in adipogenic differentiation. These results are consistent with our finding that constitutive expression of PDGF‐BB in metaphysial microvessels in physiological status favours PDGFRβ^+^ PVCs differentiating to osteoblasts, while a decrease in PDGF‐BB/PDGFRβ signalling after radiation promotes the shift of cell lineage commitment toward adipocytes.

Furthermore, we propose that radiation impairs PDGF‐BB signalling in metaphysial microvessels by regulating HIF‐1α. As a newly identified strong radio‐sensor, HIF‐1α is also the main oxygen sensor activating the transcription of genes that are involved in angiogenesis, cell survival, glucose metabolism and so forth.^63^ Previous studies revealed that PDGF‐BB was one of the downstream targets of HIF‐1α. EC‐specific *Hif1a* knockout (*Hif1a* ^*iΔEC*^) mice revealed a decline in *Pdgfb* in ECs.^18^ Besides, HIF‐1α‐siRNA significantly decreased PDGF‐BB expression in ECs, which was increased in HIF‐1α‐overexpressing ECs.^34^ Although bone is a highly vascularized tissue, the oxygen tension in bone marrow remains at a relatively low level,^64^, ^65^ especially in the metaphysis, which requires a large amount of oxygen consumption due to active osteogenesis.^65^ This may partially explain why the expression level of HIF‐1α^15^ and its downstream PDGF‐BB^15^, ^27^, ^28^ are comparatively higher in type H ECs than in type L ECs. However, weakened cellular metabolism along with widespread vascular leakage in the irradiated bone significantly reduced oxygen consumption in cells and relieved hypoxia in bone marrow.^64^ These were in line with our finding that HIF‐1α in the metaphysis and PDGF‐BB in the metaphysial ECs were both decreased after irradiation. However, ECs dysfunction caused by other factors, such as oxidative stress, is also responsible for the decline of PDGF‐BB in ECs.

Notably, *Hif1a* ^*iΔEC*^ mice revealed a reduction in type H vessels and perivascular osteoblast‐related cells in the metaphysis, including PDGFRβ^+^ PVCs, leading to a simultaneous deterioration in both angiogenesis and osteogenesis.^15^, ^18^ These appearances were similar to those occurring in the irradiated tibia in our study, implying that HIF‐1α revival in ECs may help alleviate radiation‐induced bone injury. DFM is a classic stabilizer of HIF‐1α, which interrupts the degradation of HIF‐1α by inhibiting oxygen‐dependent and iron‐dependent enzymes prolyl‐4‐hydroxylases.^66^ It can increase the accumulation and nuclear translocation of HIF‐1α to upregulate downstream angiogenic factors.^67^ A previous study showed that DFM treatment enhanced the expression of PDGF‐BB in bone marrow ECs, increased the number of type H vessels and vessel‐associated osterix^+^ cells, and ultimately increased bone mass in mice with age‐related osteoporosis.^15^ In this study, we extend the therapeutic mechanisms of DFM in radiation‐induced osteoporosis. In particular, we focussed on the HIF‐1α/PDGF‐BB/PDGFRβ axis between ECs and PVCs as well as the functional alteration of PDGFRβ^+^ cells, revealing that DFM treatment relieves bone injury by activating specific EC‐PVC crosstalk. Additionally, the alleviation of radiation‐induced bone injury by DFM is partly based on the activation of PDGFRβ^+^ PVCs. When PDGFRβ is inhibited, microvascular rarefaction and bone loss still exist.

In conclusion, the results presented in this study shed light on the diminution, disassociation and dysfunction of type H vessels and PDGFRβ^+^ PVCs in radiation‐induced bone injury. We highlight the role of HIF‐1α/PDGF‐BB/PDGFRβ signalling diminishing between type H ECs and PDGFRβ^+^ PVCs in the progression of bone damage and extend the intrinsic interaction between angiogenesis and osteogenesis. The revival of EC‐PVC crosstalk could potentially be a novel promising therapeutic candidate for bone damage with vascular problems.

## 4 MATERIALS AND METHODS

### 4.1 Mice

Wild‐type C57BL/6 mice were supplied by the Laboratory Animal Center of Sun Yat‐sen University (Guangzhou, China). Cdh5‐CreER^T2^ and Rosa26‐LSL‐Tdtomato mice were purchased from Shanghai Model Organisms Center (Shanghai, China). PDGFRβ‐CreER^T2^ mice were purchased from GemPharmatech (Nanjing, China). All animal procedures described in this study were approved by the Sun Yat‐sen University Institutional Animal Care and Use Committee. All animals were housed in the SPF barrier system facility.

PDGFRβ‐CreER^T2^;Rosa26‐LSL‐Tdtomato (PDGFRβ;Td) mice and Cdh5‐CreER^T2^;Rosa26‐LSL‐Tdtomato (Cdh5;Td) mice were generated by breeding the corresponding mouse line. All mouse lines have a C57BL/6 background. The primer sequences used in genotyping are listed in Table S1. For lineage tracing studies, mice received tamoxifen (MCE, USA) dissolved in corn oil via intra‐peritoneal injection at 4–5 weeks postnatally at a dose of 75 mg/kg every day for a total of 5 consecutive days.

### 4.2 Irradiation procedure

For the in vivo study, 8‐week to 10‐week‐old male mice were anaesthetised by inhalation of isoflurane and immobilized with fixtures. Then, both lower extremities were irradiated with laboratory irradiators (Rs2000, Rad Source, USA) at a single dose of 16 Gy (160 kV, 25 mA and 1.074 Gy/min), which is a simplified murine model to simulate clinical physical dose of 60 Gy (2 Gy/fraction). During the radiation procedure, the rest of the bodies were covered with a lead shield.

For the proliferation assay in vitro, FACS‐purified PDGFRβ;Td^+/−^ cells and HMEC‐1 (RRID:CVCL_0307) were irradiated at doses of 4, 8 or 16 Gy. For other examinations in HMEC‐1 cells, the dose was reduced to 2 Gy to avoid excessive cell death.

### 4.3 Intra‐tibial injection and intra‐peritoneal injection

The intra‐tibial injection procedure was applied according to the bone marrow transplantation protocol described previously.^31^, ^68^ Briefly, the tibia was pushed up and slightly rotated to fix the knee at ~90°. A 30 G needle was first inserted through the patellar tendon and drilled parallel along the long axis of the tibia to enter the proximal bone marrow cavity. Then, the needle was withdrawn, and a microsyringe (80401, Hamilton, USA) was carefully inserted into the drilled tunnel, and 10 μl of 100 μg/ml recombinant PDGF‐BB protein (315‐18‐50, Peprotech, USA), 50 mM IM (S1026, Selleck, USA), or equivalent volumes of PBS as placebo were slowly injected. Reagents were injected once every week and continued for 4 weeks. For irradiated mice, intra‐tibial injection was performed on days 0, 7, 14 and 21 after irradiation.

For intra‐peritoneal injection, DFM (D9533, Sigma‐Aldrich, USA) and IM were administered every other day for 21 consecutive days at doses of 100 and 70 mg/kg, respectively. For irradiated mice, the first injection was arranged on Day 7 after irradiation.

### 4.4 Micro‐computed tomography

Tibiae were dissected from WT C57BL/6 mice and fixed in 4% paraformaldehyde. Then, high‐resolution micro‐CT (μCT50, Scanco, Switzerland) was used to scan the entire length of fixed tibiae at 70 kV, 114 μA and 6 μm voxel size. After 3D reconstruction, trabecular bone quantification was performed using the manufacturer's evaluation software. For each tibia, a 1 mm region of interest was selected starting at 120 μm below the growth plate. The parameters for assessment included trabecular bone volume fraction, trabecular number, trabecular thickness, trabecular separation, structure model index and connectivity density.

### 4.5 Histological staining

Freshly dissected tibiae collected from WT C57BL/6 mice were immediately fixed in 4% paraformaldehyde overnight and decalcified in 0.5 M EDTA (pH 7.2) on shakers. Next, specimens were embedded in paraffin and prepared as longitudinally oriented sections including the metaphysis and diaphysis with a thickness of 4 μm. Paraffin slides were then used for haematoxylin and eosin staining according to the manufacturer's specifications (Servicebio, China).

### 4.6 Immunofluorescence staining

Frozen bone sections were obtained as described previously with slight modification.^69^ Briefly, freshly dissected tibiae collected from WT C57BL/6 mice or linage tracing mice were fixed immediately with ice‐cold 4% paraformaldehyde for 4 h. The fixed specimens were washed thoroughly with PBS to remove the paraformaldehyde and decalcified in 0.5 M EDTA with constant shaking at 4 °C for 48 h. After being dehydrated in 30% sucrose overnight, tibiae were embedded in the optimal cutting temperature compound (4583, Sakura, USA), and frozen sections with a thickness of 50 μm were obtained.

For immunostaining, frozen bone sections were air dried, washed with PBS for 10 min to remove optimal cutting temperature compound, permeabilized with 0.3% Triton X‐100 for 20 min, and blocked with 10% goat serum for 30 min at room temperature. Next, the sections were probed with the following primary antibodies overnight at 4°C: Endomucin (sc‐65495, Santa Cruz, USA, 1:100), eFlour660‐conjugated Endomucin (50‐5851‐82, Invitrogen, USA, 1:100), PDGFRβ (sc‐374573, Santa Cruz, 1:100), RUNX2 (12556, CST, USA, 1:200), ADIPOQ (AF6156, Beyotime, China, 1:50), TRAP (ab191406, Abcam, USA, 1:100), PDGF‐BB (sc‐365805, Santa Cruz, 1:100), HIF‐1α (20960‐1‐AP, Proteintech, USA, 1:100) and phospho‐histone H2A.X (9718, CST, USA, 1:200). After primary antibody incubation, the sections were subsequently incubated with appropriate secondary antibodies at room temperature for 1 h in the dark. Nuclei were counterstained with DAPI. Fluorescent images were acquired by laser confocal microscopy (FV3000, Olympus, Japan).

### 4.7 Flow cytometry

For flow cytometric analysis, fresh femora and tibiae were collected from WT C57BL/6 mice. Acquired bones were crushed on ice‐cold PBS and digested with 2.5 mg/ml collagenase (C0130, Sigma‐Aldrich) containing 0.2% fetal bovine serum (FBS; 10099141, Gibco, USA) at 37 °C for 20 min. After enzymatic digestion, red blood cell lysis buffer was applied, and a 70 μm filter was employed to obtain single‐cell suspensions.

For the analysis of total ECs in bone tissues, harvested cells were stained with FITC‐conjugated CD45 (103107, Biolegend, USA), FITC‐conjugated Ter119 (116205, Biolegend) and PE‐conjugated CD31 (12‐0311‐81, Invitrogen) for 30 min on ice. After washing, the cells were acquired on a flow cytometer (Cytoflex, Beckman, USA). Total ECs were quantified as CD31^+^/CD45^−^/Ter119^−^ cells using CytExpert software (Beckman, USA).

For the analysis of type H and type L ECs, harvested cells were subsequently incubated with PE‐conjugated CD31 (12‐0311‐81, Invitrogen) and eFlour660‐conjugated Endomucin (50‐5851‐82, Invitrogen). Type H ECs were quantified as CD31^high^/Emcn^high^, while type L ECs were quantified as CD31^low^/Emcn^low^.^15^

For the analysis of PDGFRβ^+^ cells, the metaphysis regions of tibiae were crushed and digested with collagenase for 1 h, and the harvested cells were additionally labelled with FITC‐conjugated CD45, FITC‐conjugated Ter119, PE‐conjugated CD31 and APC‐conjugated CD140b (17‐1402‐80, Invitrogen). PDGFRβ^+^ cells were defined as CD45^−^/Ter119^−^/CD31^−^/CD140b^+^ cells.

### 4.8 In vitro isolation and culture of PDGFRβ;Td^+^ cells

Fresh femora and tibiae from nonirradiated or irradiated PDGFRβ;Td mice were thoroughly dissected from soft tissues. Then the metaphysis regions were reserved and cut into small pieces in ice‐cold PBS under a sterilized environment. The harvested samples were then washed with PBS twice and incubated with 2.5 mg/ml collagenase dissolved in alpha‐modified Eagle's medium (α‐MEM, C12571500BT, Gibco) at 37°C for 1 h under constant shaking. After enzyme digestion, the supernatants were removed by centrifugation, and the bone fragments and cell pellets were re‐suspended in α‐MEM with 10% FBS and 1% penicillin/streptomycin (P/S, 15140122, Gibco) and transferred carefully to culture dishes. Cells were incubated in a humidified environment at 37°C with 5% carbon dioxide. After 48 h, the culture medium was replaced gently to remove the floating cells. Follow‐up replacement of the medium was performed every 2–3 days. When the adherent cells spread to more than 90% of the bottom, they were digested with 0.25% trypsin–EDTA solution (25200072, Gibco) and harvested for FACS. Cell sorting was performed with a BD FACSAria Fusion Flow Cytometer (BD Bioscience) and analysed using BD FACSDiva software. Purified PDGFRβ;Td^+^ cells were then re‐suspended in α‐MEM complete culture medium and inoculated in pore plates for subsequent proliferation, migration and differentiation experiments.

To evaluate of the effect of PDGF‐BB/PDGFRβ signalling on PDGFRβ^+^ cells, 10 ng/ml recombinant PDGF‐BB protein, 1 μg/ml anti‐PDGF‐BB neutralizing antibody (AF‐220‐SP, R&D System, USA), and 5 μM IM were used during the culture period.

### 4.9 Isolation and cell sorting of Cdh5;Td^+^ ECs

Fresh femora and tibiae from Cdh5;Td mice were processed to obtain single‐cell suspensions as described in the FCM section. After FACS, purified Cdh5;Td^+^ ECs were centrifuged and used for total RNA isolation.

### 4.10 In vitro culture of microvascular ECs

HMEC‐1 cells were cultured in an EC medium (1001, ScienCell, USA) containing 5% FBS, 1% endothelial cell growth supplement (ECGS) and 1% P/S according to the manufacturer's instructions. HMEC‐1 cells in the irradiation group received 2 Gy x‐ray radiation and were followed by another 14‐day culture period. For the DFM treatment group, HMEC‐1 cells were incubated with 5 μM DFM for 7 days before conditioned medium collection, total RNA isolation, and co‐culture with PDGFRβ;Td^+^ cells on Matrigel.

### 4.11 BMS collection

BMS from tibiae were collected as previously described with slight modification^27^, ^31^ and used for cell migration assays and enzyme‐linked immunosorbent assay (ELISA) analysis. Briefly, dissected tibiae from WT C57BL/6 mice were cleaned to remove adherent soft tissues in ice‐cold PBS, and the bone marrow was exposed by carefully cutting off the distal end. Then, specimens were placed in 1.5 ml Eppendorf tubes containing 100 μl of ice‐cold PBS with the distal end oriented toward the bottom of the tube. After centrifugation at 3000 rpm at 4°C for 20 min, 70 μl of total supernatant was collected and stored at −80 °C.

### 4.12 Conditioned medium collection

The conditioned medium (CM) of HMEC‐1 cells was collected for cell migration assays and ELISA analysis. Equivalent volumes of CM in different groups with an approximate number of cells were collected. Supernatants were centrifuged at 3000 rpm at 4°C for 10 min and carefully transferred to clean 1.5 ml Eppendorf tubes for immediate use or stored at −80°C.

### 4.13 Enzyme‐linked immunosorbent assay

PDGF‐BB analysis of BMS or CM was performed using a QuantiCyto® PDGF‐BB ELISA kit (EMC032.96, Neobioscience, China) based on the manufacturer's instructions.

### 4.14 Cell proliferation assay

FACS‐purified PDGFRβ;Td^+^ cells, PDGFRβ;Td^−^ cells or HMEC‐1 cells were seeded at a density of 5 × 10^3^ cells/well in 96‐well plates. Cell Counting Kit‐8 reagent (CK04, Dojindo, Japan) was used to measure cellular proliferation at Days 1, 4 and 7. Absorbance at 450 nm was measured under a microplate reader (Infinite 200 PRO, Tecan, Switzerland).

### 4.15 Cell migration assay

The chemotactic effect of BMS from tibiae and CM from HMEC‐1 cells on PDGFRβ;Td^+^ cells was evaluated by a cell migration assay. In brief, PDGFRβ;Td^+^ cells were seeded in the upper chambers of the cell‐cultured insert with 8 μm pore filters (353097, Falcon, USA) at a concentration of 1 × 10^4^ cells/well, and BMS or CM with recombinant PDGF‐BB protein or anti‐PDGF‐BB neutralizing antibody was added to the lower chambers. In the IM treatment group, PDGFRβ;Td^+^ cells were pre‐incubated with IM for 1 h to block PDGFRβ before cell seeding. After 12 h, the migrated cells were fixed in 4% paraformaldehyde, stained with crystal violet, captured under an inverted microscope system (Axio Observer 5, Zeiss, Germany), and quantified using ImageJ software.

### 4.16 Osteogenic and adipogenic differentiation assay

In the osteogenic differentiation experiment, PDGFRβ;Td+ cells were induced for 7 days for ALP staining (C3206, Beyotime) and ALP activity evaluation (A059‐2‐2, Jian Cheng Technology, China), 14 days for Western blot assays, and 21 days for Alizarin Red staining (ALIR‐10001, Cyagen) unless otherwise specified. Assays were performed according to the manufacturer's instructions. Semiquantitative analysis of matrix mineralization was detected using cetyl‐pyridinium chloride (CPC; C9002, Sigma–Aldrich), and absorbance at 562 nm was measured with a microplate reader.

In the adipogenic differentiation experiment, PDGFRβ;Td+ cells were induced for 14 days for Western blot assays and Oil red O staining (OLIR‐10001, Cyagen). Assays were performed according to the manufacturer's instructions.

### 4.17 Quantitative real‐time PCR

For analysis of the mRNA expression levels, total RNA was isolated using an RNA Quick Purification kit (RN001, ESscience, China) according to the manufacturer's protocol. A total of 500 ng RNA per reaction was used to generate cDNA with Hifair® III first Strand cDNA Synthesis SuperMix (11141, Yeasen, China). Quantitative real‐time PCR (qRT‐PCR) was performed using the Hieff® qPCR SYBR Green Master Mix (11201, Yeasen) on a Roche LightCycler® 96 Real‐Time PCR System, according to the manufacturer's instructions. Gene expression was normalized to that of *Actb* as a standard. The primer sequences used in this study are listed in Table S2.

### 4.18 Western blot

Western blot analysis was performed as previously described.^22^ In particular, for evaluation of the protein translation level in whole bone tissues, dissected tibiae were crushed finely in ice‐cold PBS and digested with collagenase before protein extraction. The primary antibodies used in this study included Collagen1 (COL1, AF7001, Affinity, USA, 1:1000), RUNX2 (12556, CST, 1:1000), Osteocalcin (OCN, sc‐390877, Santa Cruz, 1:1000), PPARγ (sc‐7273, Santa Cruz, 1:1000), ADIPOQ (AF6156, Beyotime, 1:1000), PDGFRβ (sc‐374573, Santa Cruz, 1:1000), pPDGFRβ (sc‐365464, Santa Cruz, 1:1000), PDGF‐BB (sc‐365805, Santa Cruz, 1:1000), β‐Actin (8457, CST, 1:1000), HIF‐1α (20960‐1‐AP, Proteintech, USA, 1:1000) and β‐Tubulin (2146, CST, 1:1000). Protein bands were visualized using Immobilon ECL Ultra Western HRP Substrate (Millipore, USA), and images were collected by a ChemiDoc imaging system (Bio‐Rad, USA).

### 4.19 Osteo‐angiogenic affinity assay

The co‐culture of PDGFRβ;Td^+^ cells and HMEC‐1 cells on Matrigel (356234, Corning, USA) was arranged to detect the recruitment effect of ECs on PDGFRβ;Td^+^ cells as previously described with slight modification.^20^ Briefly, HMEC‐1 cells were labelled with DiO (green) membrane dye (C1993S, Beyotime) and seeded at a concentration of 2 × 10^4^ cells/well on Matrigel‐pre‐coated 96‐well plates. After incubation for 1 h, 2 × 10^3^ cells/well of PDGFRβ;Td^+^ cells (red) were added and co‐cultured with endothelial tubules for 3 h. In the IM treatment group, PDGFRβ;Td^+^ cells were pre‐incubated with 5 μM IM for 1 h. Images were taken by an inverted microscope system and analysed using ImageJ software. The endothelium‐related cells were calculated by counting Td^+^ cells on tubules per field of view.

### 4.20 Statistical analyses

All data are presented as the mean ± SD. Unpaired, two‐sided Student's *t*‐test was used for comparisons between two treatment groups, and one‐way analysis of variance with Tukey's post hoc test was used for multiple comparisons. Differences were considered significant at *p* < 0.05. GraphPad Prism 8 (GraphPad Software, USA) was employed for statistical analysis.

## AUTHOR CONTRIBUTIONS

Jiayan Li, Xiaodan Chen, Bin Cheng and Juan Xia conceived the study and designed the experiments. Jiayan Li, Xiaodan Chen and Lin Ren performed the experiments, acquired the data and analysed the results. Xijuan Chen, Tong Wu, Yun Wang and Xianyue Ren provided technical consultation and contributed to data interpretation. Jiayan Li, Xiaodan Chen, Bin Cheng and Juan Xia prepared the article. Jiayan Li and Xiaodan Chen contributed equally to this work.

## CONFLICT OF INTEREST

The authors have declared that no conflict of interest exists.

## Supporting information


**Data S1:** Supporting information

## Data Availability

The data that support the findings of this study are available from the corresponding author upon reasonable request.
