# Supplementary material for: Type H vessel/platelet‐derived growth factor receptor β+ perivascular cell disintegration is involved in vascular injury and bone loss in radiation‐induced bone damage
Source: Cell Prolif. 2023 Jan 24;56(7):e13406. doi: 10.1111/cpr.13406 (PMC10334283; doi:10.1111/cpr.13406)
Supplement: Supplementary file 1 — Data S1: Supporting information [file CPR-56-e13406-s001.docx]

Supplementary materials

Figure S1


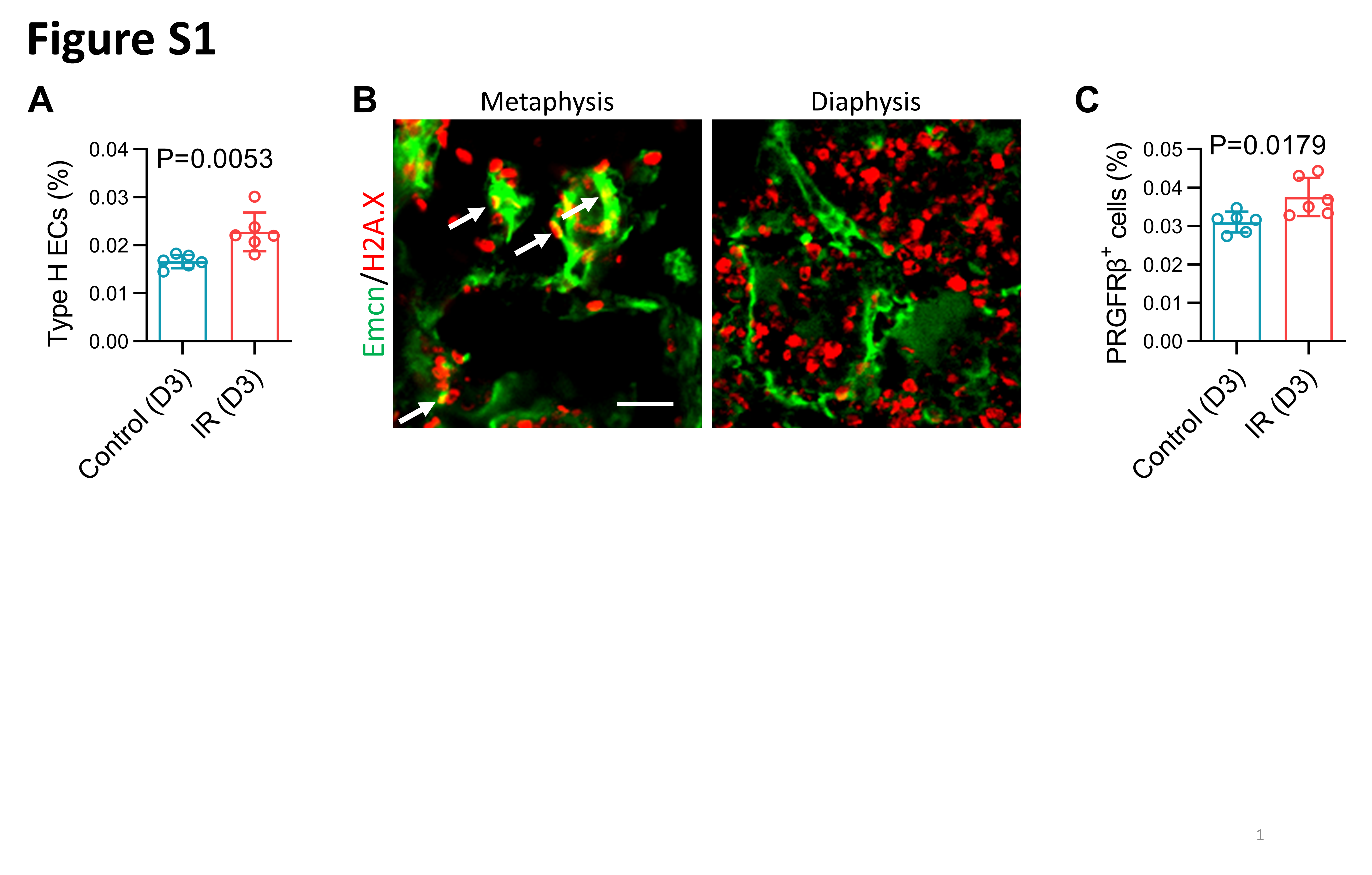


**Figure S1 Early-phase reaction of type H ECs and PDGFRβ PVCs after irradiation.**

**(A)** Flow cytometric quantitation of type H ECs (CD31^high^/Emcn^high^) from control mice and mice 3 days post-irradiation. (n=6). **(B)** Representative double-staining images of Emcn (green) and H2A.X (red) in tibial sections at 3 days post-irradiation. The white arrows represent H2A.X-positive ECs. Scale bars, 25 μm. **(C)** Flow cytometric quantitation of PDGFRβ^+^ cells (CD45^−^/Ter119^−^/CD31^−^/CD140b^+^) from mice of control and 3 days post-irradiation. (n=6). Data are represented as the mean ± S.D. The *P* value was calculated by unpaired, two-tailed Student’s *t*-test.

Figure S2


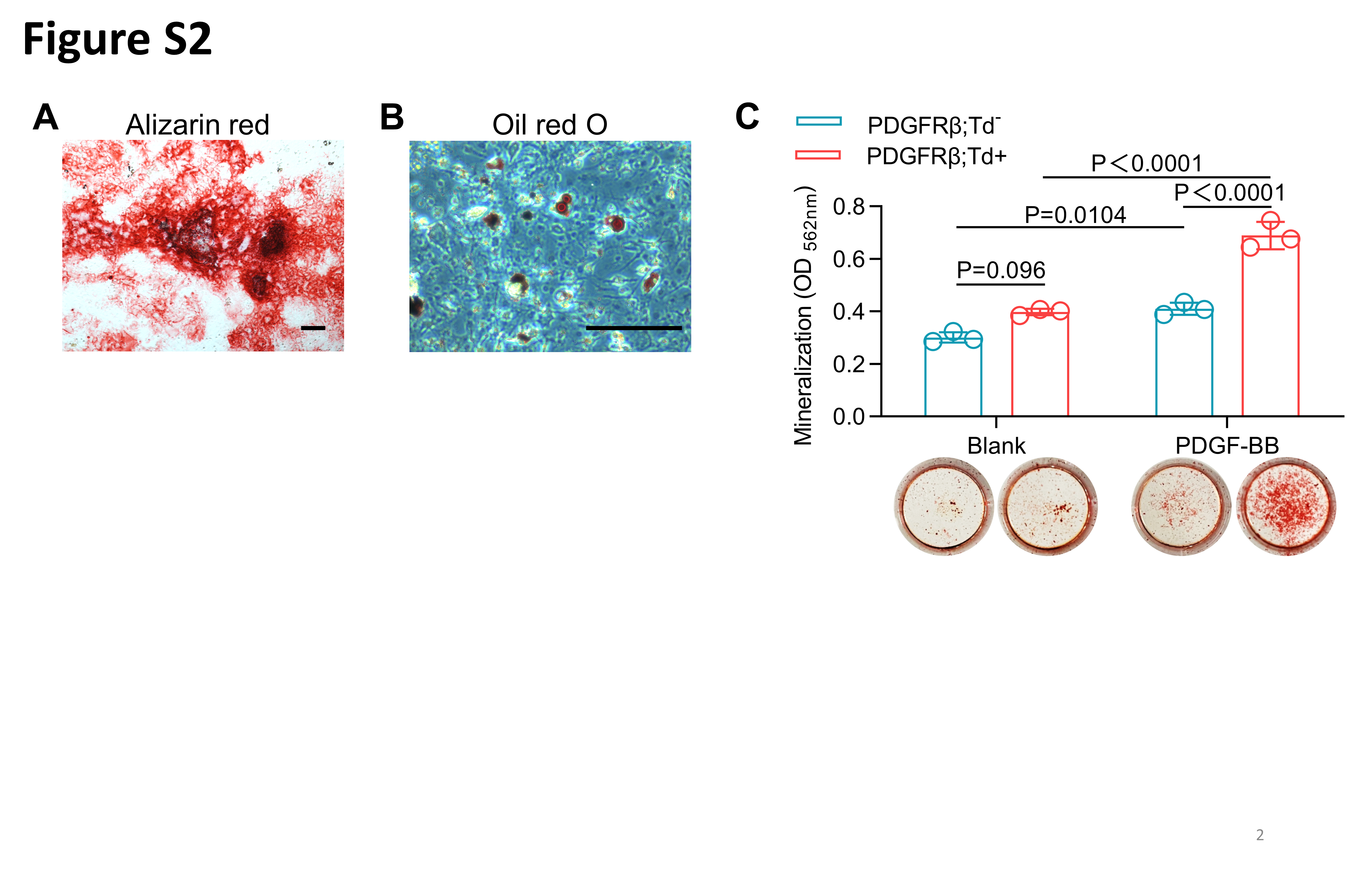


**Figure S2. PDGFRβ;Td^+^ cells display superior osteogenic differentiation potential.**

**(A, B)** Representative Alizarin red staining (A) and Oil red O staining (B) images of FACS-purified PDGFRβ;Td^+^ cells after *in vitro* osteogenic and adipogenic induction, respectively. Scale bars, 200 μm. **(C)** Representative Alizarin red staining images and semiquantitative analysis of PDGFRβ;Td^-^positive and PDGFRβ;Td^-^negative subsets after 14 days of osteogenic induction with or without exogenous PDGF-BB. (n=3). Scale bars, 200 μm. Data are represented as the mean ± S.D.The *P* value was calculated by two-way ANOVA with Tukey’s post-hoc test.

Figure S3


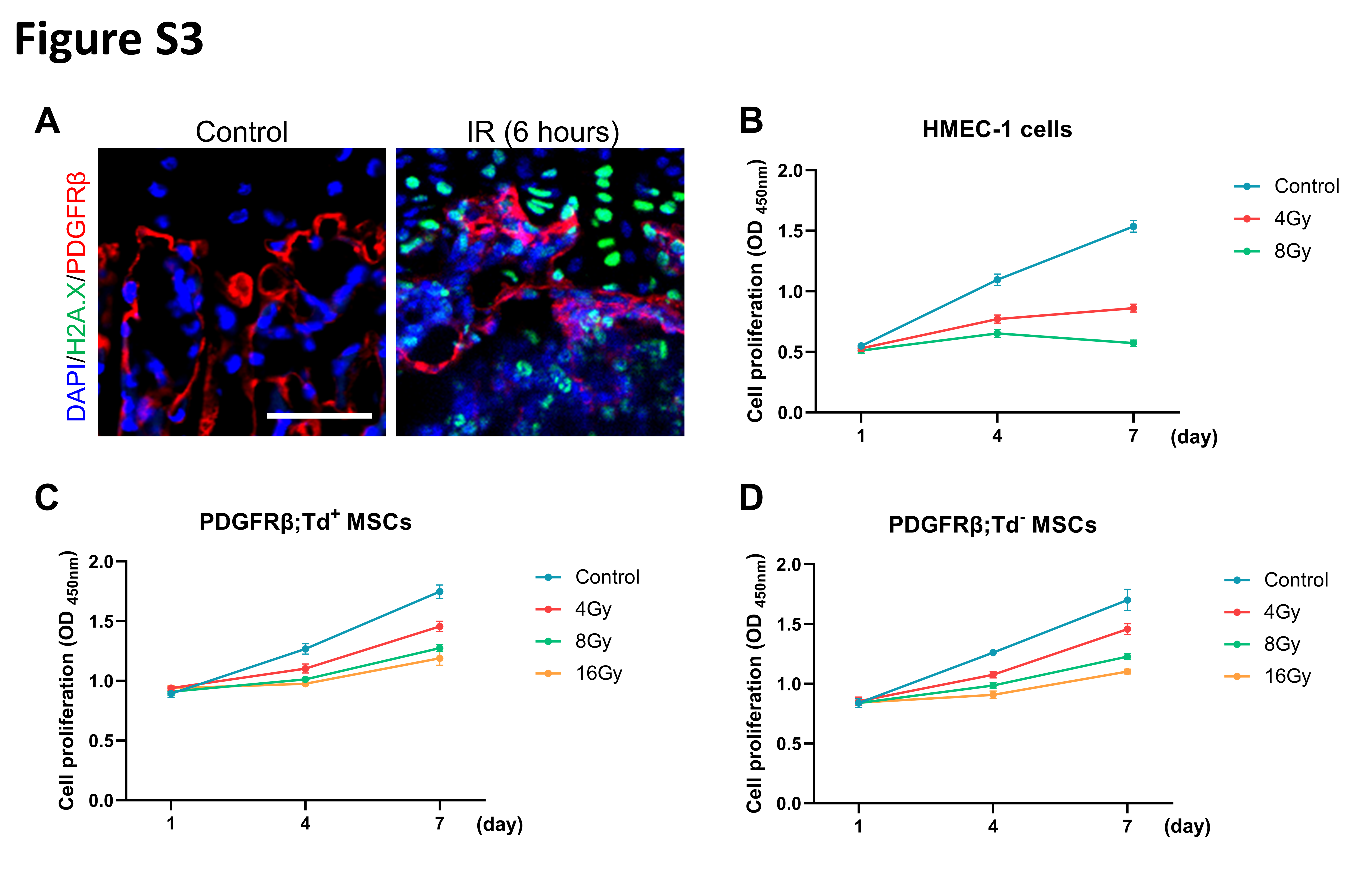


**Figure S3. PDGFRβ^+^ cells show relative resistance to direct radiation exposure.**

**(A)** Representative H2A.X (green) and PDGFRβ(red) double-staining images of tibial sections from mice of control and 3 days post-irradiation. Nuclei, DAPI (blue). Scale bars, 10 μm. **(B)** CCK-8 assay showed the proliferation ability of HMEC-1 cells after direct radiation exposure *in vitro*. (n=4). **(C, D)** CCK-8 assay showed the proliferation ability of the PDGFRβ-positive subset (C) and the PDGFRβ-negative subset (D) after direct radiation exposure *in vitro*. (n=4). Data are represented as the mean ± S.D.

Figure S4


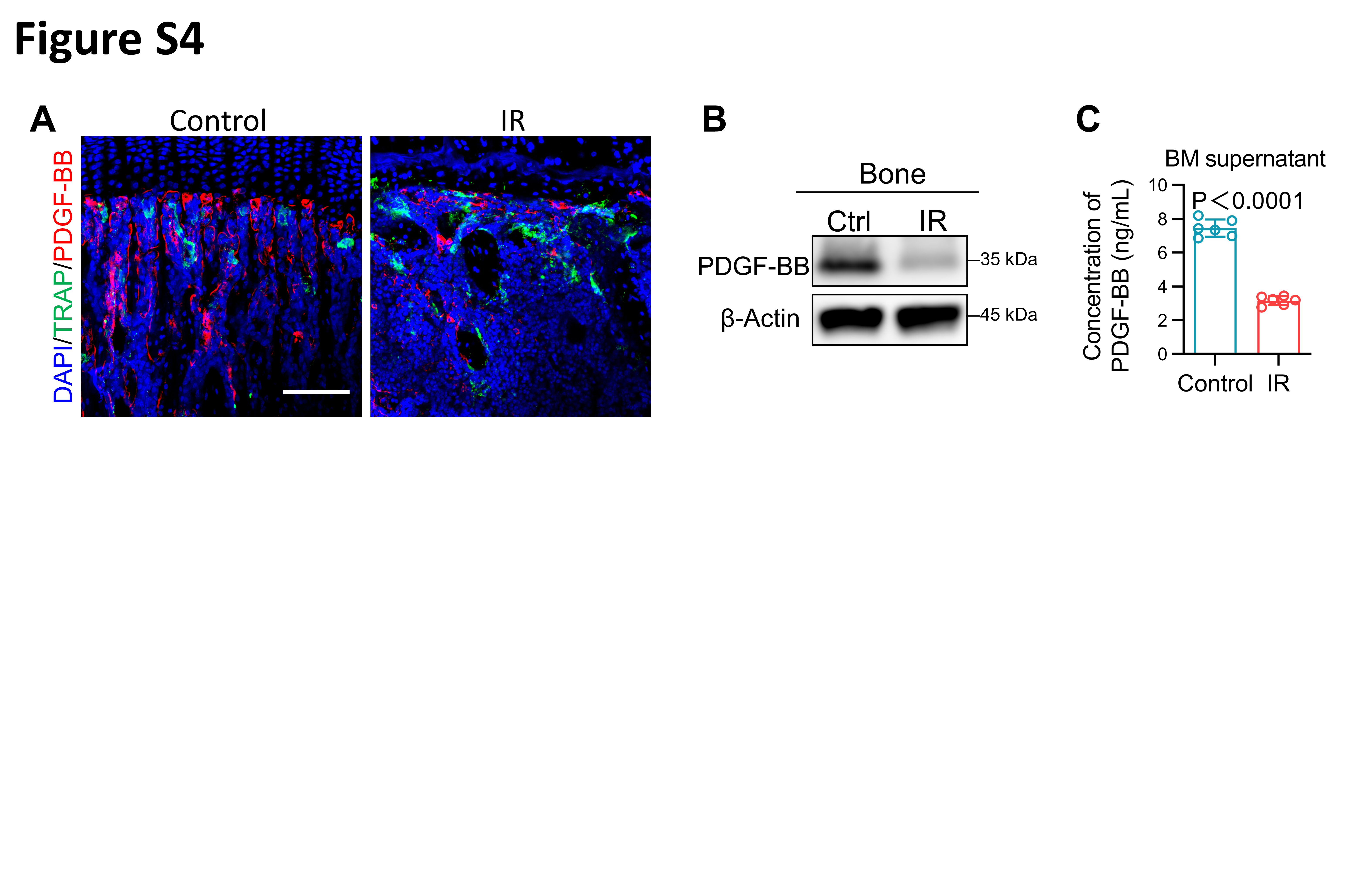


**Figure S4. PDGF-BB declines in bone marrow after irradiation.**

**(A)** Representative fluorescence images of the control and irradiated tibial sections stained with TRAP (green), PDGF-BB (red), and DAPI (blue). Scale bars, 50 μm. **(B)** Western blot analysis of PDGF-BB in whole bone tissues of the control and irradiated tibiae. **(C)** ELISA analysis of PDGF-BB in bone marrow (BM) supernatants collected from the control and irradiated mice. (n=6). Data are represented as the mean ± S.D. The *P* value was calculated by unpaired, two-tailed Student’s *t*-test.

Figure S5

**
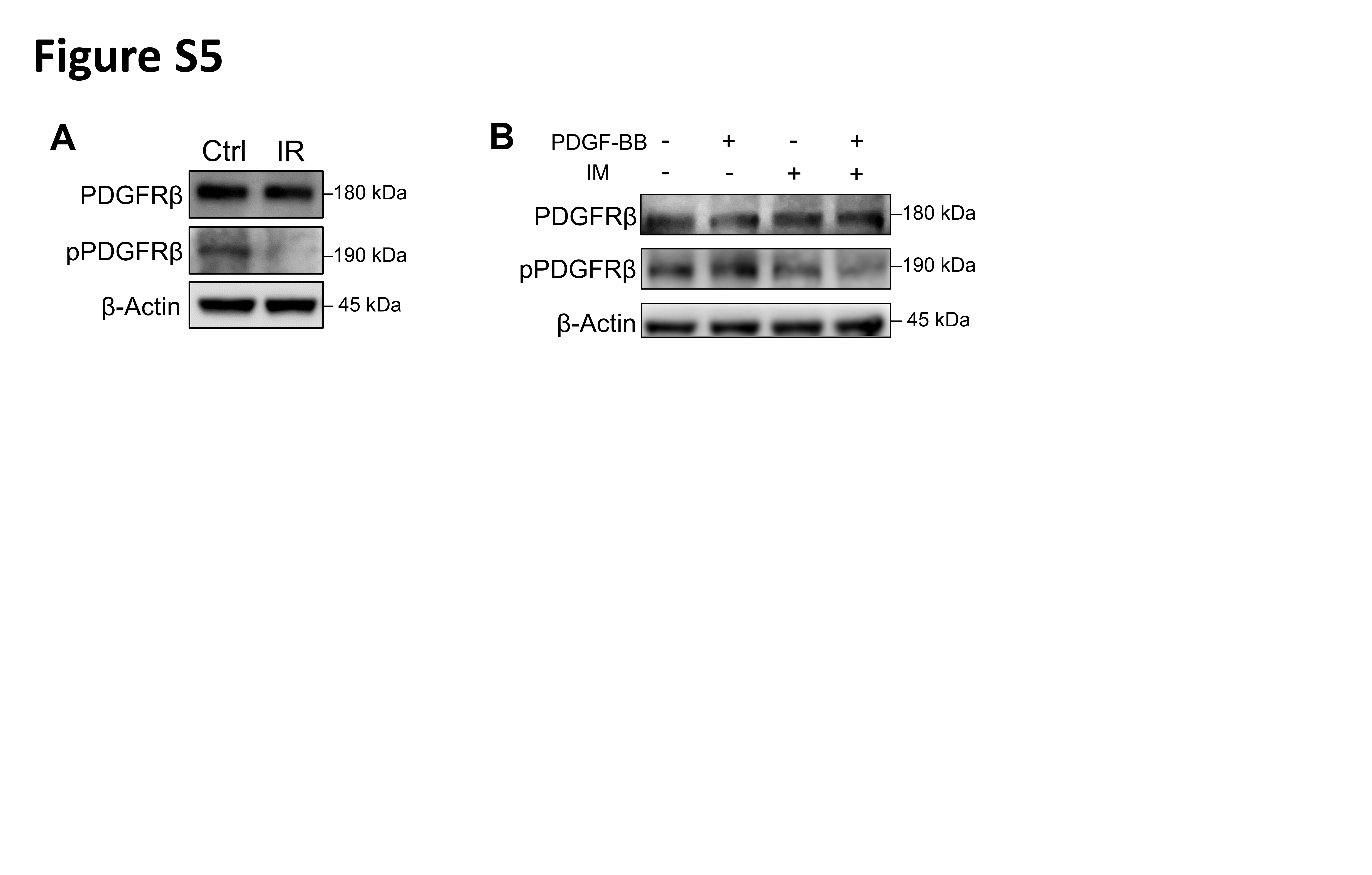
**

**Figure S5 Radiation downregulated the phosphorylation level of PDGFRβ in PDGFRβ;Td^+^ cells.**

**(A)** Western blot analysis of the phosphorylation level of PDGFRβ in PDGFRβ;Td^+^ cells isolated from the control and irradiated mice. **(B)** Western blot analysis of the phosphorylation level of PDGFRβ in PDGFRβ;Td^+^ cells treated with PDGF-BB and/or imatinib mesylate (IM) *in vitro*.

Figure S6

**
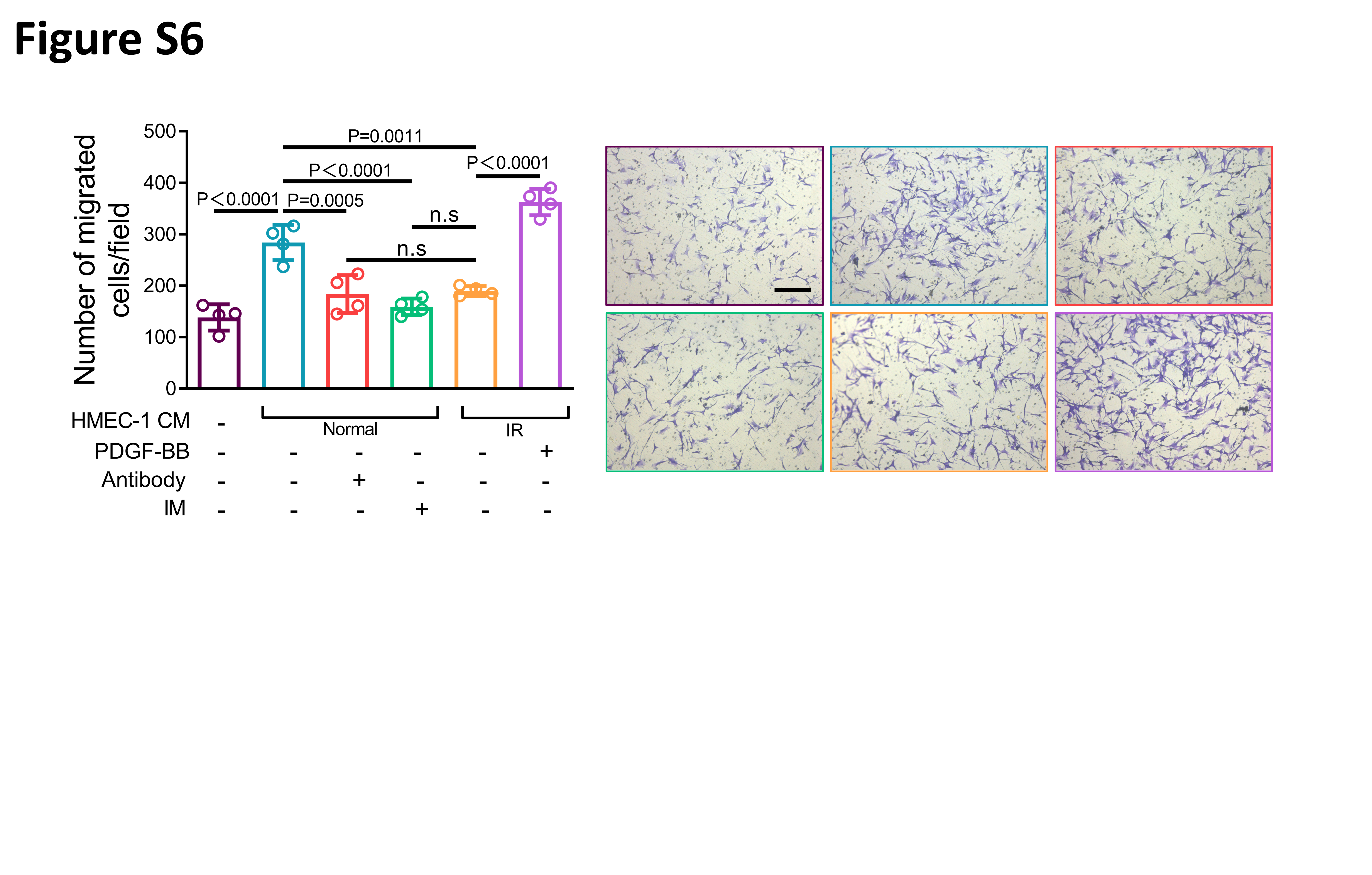
**

**Figure S6. EC-derived PDGF-BB regulates the migration of PDGFRβ;Td^+^ cells *in vitro*.**

Migration assay of PDGFRβ;Td^+^ cells in the conditioned medium (CM) of HMEC-1 cells. Td^+^ cells received the additional intervention with recombinant PDGF-BB protein, PDGF-BB neutralizing antibody, or IM. Scale bars, 100 μm. (n=4). Data are represented as the mean ± S.D. The *P* value was calculated by one-way ANOVA with Tukey’s post-hoc test.

Figure S7

**Figure S7. PDGF-BB treatment increased collagen deposition in the irradiated tibiae.**

Representative Masson staining images of the irradiated tibiae with or without PDGF-BB treatment. The blue-stain regions represent collagen. Scale bars, 300 μm.

Figure S8


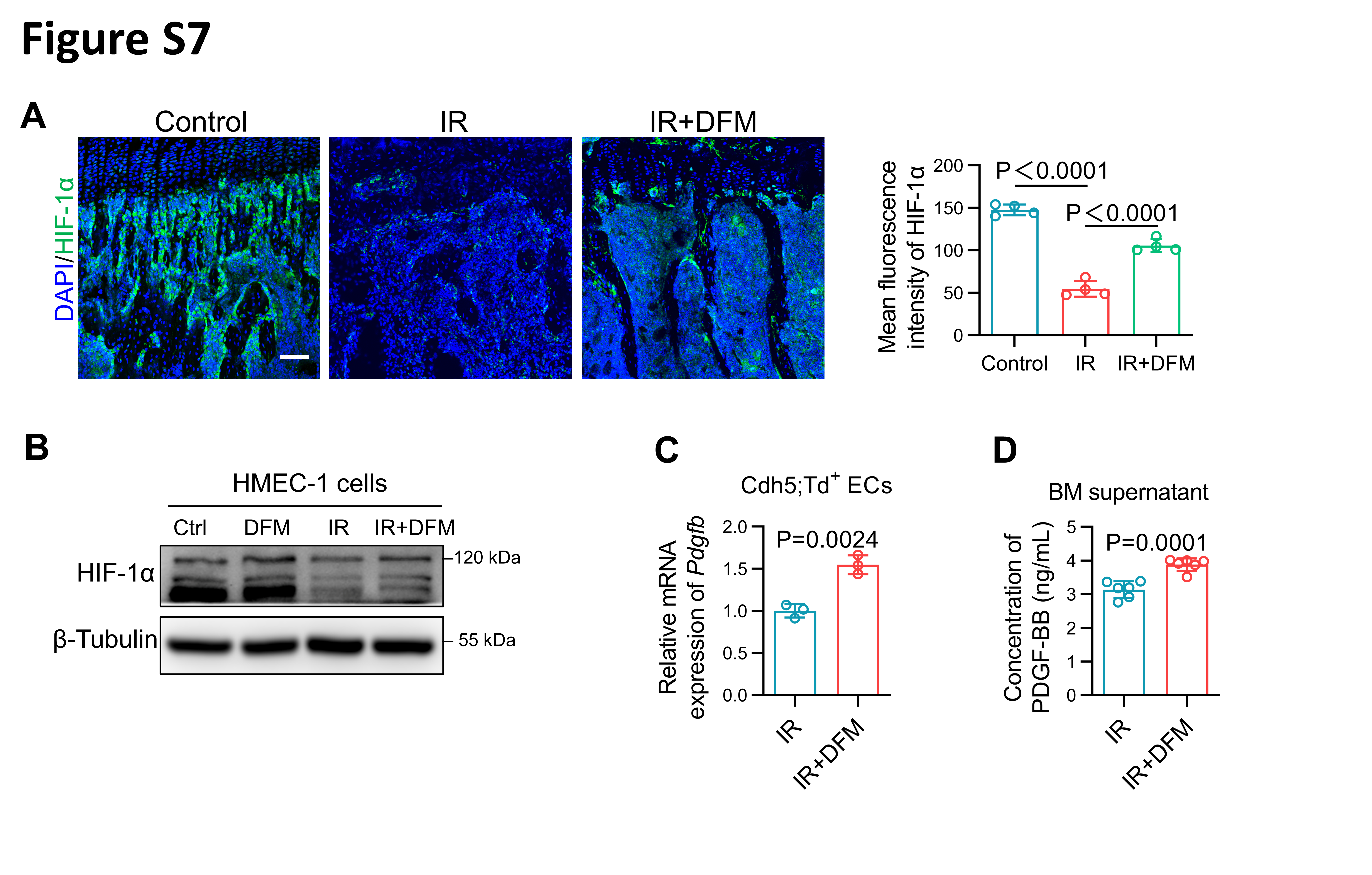


**Figure S8 DFM reverses the downregulation of the HIF-1α/PDGF-BB axis after irradiation.**

**(A)** Representative HIF-1α (green) staining images of the metaphysis regions. Nuclei, DAPI (blue). Quantification of fluorescence intensity of HIF-1α. Scale bars, 100 μm. (n=4). **(B)** Western blot analysis of HIF-1α in HMEC-1 cells. **(C)** qRT‒PCR analysis of the *Pdgfb* gene in Cdh5;Td^+^ ECs from irradiated mice with or without DFM treatment (n=3). **(D)** ELISA analysis of PDGF-BB in bone marrow (BM) supernatant from irradiated mice with or without DFM treatment. (n=6). Data are represented as the mean ± S.D. The *P* value was calculated by unpaired, two-tailed Student’s *t*-test when comparing two treatment groups and by one-way ANOVA with Tukey’s post-hoc test when comparing multiple groups.

**Table S1. Primer sequences used in genotyping.**

| **Gene** | **Primer Type** | **Sequence (5’→3’)** |
| --- | --- | --- |
| Rosa26-LSL-Tdtomato | Tdtomato-Wt-F | AAGGGAGCTGCAGTGGAGTA |
|  | Tdtomato-Wt-R | CCGAAAATCTGTGGGAAGTC |
|  | Tdtomato-Ki-F | GGCATTAAAGCAGCGTATCC |
|  | Tdtomato-Ki-R | CTGTTCCTGTACGGCATGG |
| Cdh5-CreER^T2^ | Cdh5-Wt-F | GAGGAGGGCGGTGGTGAGATGGA |
|  | Cdh5-Wt-R | GAAGGGGCGCTGGGTTGAAGAGTC |
|  | Cdh5-Ki-F | GGCTACGAGGGCGCAGAGTCCAT |
|  | Cdh5-Ki-R | CCGCCGCATAACCAGTGAAACAGC |
| PDGFRβ-CreER^T2^ | Pdgfrb-Ki-F | AGCTCCAAGAAGAGCCACAGC |
|  | Pdgfrb-Ki-R | TCCGGTTATTCAACTTGCACCATGC |
|  | Pdgfrb-Wt-F | AGCTCCAAGAAGAGCCACAGC |
|  | Pdgfrb-Wt-R | AGTGTAGCTGCTGGGAGGCCA |

**Table S2. Primer sequences used for qRT‒PCR.**

| **Gene** | **Primer Type** | **Sequence (5’→3’)** |
| --- | --- | --- |
| *hPDGFB* | Forward | CTCGATCCGCTCCTTTGATGA |
|  | Reverse | CGTTGGTGCGGTCTATGAG |
| *hACTB* | Forward | CTCGCCTTTGCCGATCC |
|  | Reverse | TCTCCATGTCGTCCCAGTTG |
| *mPdgfb* | Forward | CATCCGCTCCTTTGATGATCTT |
|  | Reverse | GTGCTCGGGTCATGTTCAAGT |
| *mActb* | Forward | GGCTGTATTCCCCTCCATCG |
|  | Reverse | CCAGTTGGTAACAATGCCATGT |
